# Current State of Evidence for Implant Placement and Loading in Partially Edentulous Patients: A Systematic Review

**DOI:** 10.1111/cid.70120

**Published:** 2026-01-23

**Authors:** German O. Gallucci, Adam Hamilton, Samuel Akhondi, Kevser Pala, Juan Francisco Peña‐Cardelles

**Affiliations:** ^1^ Department of Restorative Dentistry and Biomaterials Sciences Harvard School of Dental Medicine Boston Massachusetts USA; ^2^ Division of Oral Restorative and Rehabilitative Sciences University of Western Australia Perth Western Australia Australia

**Keywords:** dental implants, early loading, early placement, immediate loading, immediate placement

## Abstract

**Objectives:**

To systematically review the updated evidence for the clinical outcome of fixed implant prostheses treated with different combinations of implant placement and loading protocols in partially edentulous patients.

**Materials and Methods:**

An electronic search was performed in Medline, Embase, and Central to identify studies of implants subjected to immediate placement + immediate restoration/loading (Type 1A), immediate placement + early loading (Type 1B), immediate placement + conventional loading (Type 1C), early placement + immediate restoration/loading (Type 2–3A), early placement + early loading (Type 2–3B), early placement + conventional loading (Type 2–3C), late placement + immediate restoration/loading (Type 4A), late placement + early loading (Type 4B), late placement + conventional loading (Type 4C) with implant‐supported fixed dental prostheses (IFDPs) in partially edentulous patients. A cumulative survival rate for each type of the implant placement and loading protocols was weighted by the duration of follow‐up and number of implants.

**Results:**

From 11 427 records, 140 studies (42 RCTs; 98 CCTs/cohort studies) encompassing 10 456 implants met the criteria. Weighted cumulative survival rates for each protocol were: 98.0% (Type 1A), 91.6% (Type 1B), 95.0% (Type 1C), 97.8% (Type 2–3A), 100% (Type 2–3B), 94.0% (Type 2–3C), 97.2% (Type 4A), 97.9% (Type 4B), 97.5% (Type 4C). Protocols 1A, 1C, 2–3C, 4A, 4B, and 4C satisfy scientific and/or clinical validation thresholds, whereas 1B and 2–3B remain insufficiently documented despite high numeric survival.

**Conclusions:**

In immediate placement, Type 1C shows strong survival rates. It is considered scientifically and clinically validated, while Type 1A also meets the criteria for a scientifically and clinically validated protocol with high survival rates. Meanwhile, Type 1B continues to show lower and more variable survival rates—being clinically documented—underscoring the need for careful case selection. Regarding early placement, Type 2–3C is recognized as a scientifically and clinically validated protocol. Type 2–3A, which was previously underreported, now demonstrates similarly validated survival rates that expand the evidence for early implant placement with immediate loading. Although Type 2–3B is clinically documented, it still lacks sufficient evidence. All late implant placement protocols are considered scientifically and clinically validated: Type 4C offers high survival with long‐term predictability, while Type 4A and Type 4B maintain stable survival rates backed by well‐established evidence.

## 1 Introduction

The selection of an appropriate implant placement and loading protocol is a key treatment planning consideration which can influence the outcome. Since the early developments in oral implantology, various surgical and prosthodontic approaches have been explored based on the biological principles of wound healing and osseointegration as well as clinical trials which aim to optimize osseointegration, minimize treatment time, and improve functional and esthetic outcomes.

The evolution of these protocols has been extensively analyzed, with a key concept emerging in 2018 that recognized implant placement and implant loading protocols as an interdependent event which should be considered concurrently. This led to the development of a new implant placement and loading classification system combining the various implant placement and loading protocols [1]. Recent systematic reviews and meta‐analyses emphasize the clinical viability of immediate implant placement and immediate loading in suitable sites, reinforcing previous recommendations on their effectiveness and long‐term outcomes [2, 3, 4, 5].

Implant placement protocols are classified into immediate (Type 1), early with soft tissue healing (Type 2), early with partial bone healing (Type 3), and late after complete bone healing (Type 4) [6, 7, 8]. Loading protocols follow a similar categorization: immediate (within 1 week), early (1 week to 2 months), and conventional (after 2 months). While immediate and early placement can reduce treatment time and enhance patient satisfaction, their success depends on anatomical and biological factors such as alveolar ridge condition, primary stability, and soft and hard tissue integrity [9, 10, 11].

Recent systematic reviews and meta‐analyses have provided updated insights into implant placement and loading strategies. The latest ITI Consensus Report emphasized the importance of patient‐specific selection criteria in immediate placement and loading protocols, particularly in the anterior maxilla, where esthetic concerns are paramount [4]. Studies have shown that while immediate loading offers high patient satisfaction and shorter treatment times, it requires meticulous case selection, including sufficient buccal bone thickness and primary stability to mitigate risks such as mid‐facial recession and papilla height loss [2, 3, 5]. A systematic review analyzing implant survival in different locations highlighted that while immediate placement and loading yield favorable outcomes in the anterior maxilla, late placement remains the gold standard for the posterior mandible, where bone density and primary stability considerations differ [12]. Furthermore, a comprehensive review of implant loading protocols for edentulous patients reaffirmed that immediate and early loading are viable approaches when certain clinical criteria are met, such as achieving sufficient insertion torque, high resonance frequency values, using surface‐modified implants, and performing careful patient selection to ensure adequate bone quality and quantity. However, conventional loading still demonstrates the highest long‐term predictability in complex cases [13].

Recent advancements in surgical techniques, implant surface modifications, and digital planning have contributed to more predictable outcomes across different placement and loading strategies. This updated systematic review builds upon previous findings by Gallucci et al. [1], incorporating a larger dataset and extended follow‐up periods to evaluate implant survival and success rates across multiple protocols. While the 2018 review was limited by a smaller number of included studies and shorter follow‐up durations, the present work addresses these gaps by integrating a broader and more contemporary evidence base with extended observation periods, providing a more comprehensive assessment of clinical outcomes. The increased number of studies allows for a review of the scientific and clinical validation of the various implant placement and loading protocols to assist guiding clinicians in decision making with evidence‐based implant treatments.

This review aims to provide updated clinical insights into the survival and success rates of different implant placement and loading protocols in partially edentulous patients.

## 2 Materials and Methods

This systematic review was performed following methodological standards and guidelines, including the Preferred Reporting Items for Systematic Reviews and Meta‐Analyses (PRISMA) developed by Liberati et al. [14], the Standards for Developing Trustworthy Clinical Practice Guidelines issued by the Institute of Medicine (IOM) [15], and the Cochrane Handbook for Systematic Reviews of Interventions authored by Higgins and Green in 2017 [16]. This review was conducted as an update of a previously registered systematic review (PROSPERO ID: CRD42017062420) [1], following the same predefined methodology and eligibility criteria.

### 2.1 Focus Question

The PICO question was comprised of the following parameters: Population encompassed partially edentulous patients; intervention involved immediate/early placement and loading protocols; comparison intervention consisted of late placement and conventional loading protocols; and outcome focused on implant‐prosthodontic survival and success. This resulted in the following PICO question: “In partially edentulous patients with immediate or early placement and loading protocols, do the implant‐prosthodontic survival and success differ when compared to conventional protocols?”

Implant placement protocols were categorized based on existing literature: immediate placement referred to same‐day placement post‐extraction; early placement involved timing between 4 and 8 weeks (soft tissue healing) or 12 and 16 weeks (partial bone healing); late placement occurred after full bone healing exceeding 6 months [6, 7, 8].

Loading was classified as immediate (within 1 week), early (between 1 week and 2 months), or conventional (after 2 months), considering only functional loading.

This classification aligned with criteria from previous ITI Consensus Conferences [4, 13, 17, 18, 19, 20, 21, 22, 23, 24, 25, 26, 27].

### 2.2 Search Strategy

An electronic literature search was carried out in PubMed/MEDLINE, Embase, and the Cochrane CENTRAL database for English‐language articles published up to January 1, 2024.

In the PubMed/MEDLINE search, controlled vocabulary (MeSH terms) was combined with keywords whenever applicable. The specific terms used in the search were: (dental implantation, endosseous[MeSH] OR dental implants[MeSH] OR implantation OR implant OR implants) AND (denture, partial, fixed[MeSH] OR dental prostheses, implant‐supported[MeSH] OR fixed partial denture OR FPD OR FPDs OR fixed dental prosthesis OR fixed dental prostheses OR bridge OR crown) AND (immediate implant OR immediate implantation OR immediate implant placement OR immediate placement OR immediate OR early OR placement OR time OR timing OR fresh extraction sockets OR immediate extraction sockets OR post‐extraction implant placement OR post‐extractive OR early implantation OR early implant placement) AND (immediate dental implant loading[MeSH] OR function OR time OR immediate OR early OR load) AND (English[Language]).

The references were managed with a specific bibliographic software (EndNote X8, version 8.1, Thomson Reuters, New York, NY, USA).

### 2.3 Selection Criteria

Studies of any design were considered eligible as long as they fulfilled the following criteria:

- Human studies;
- At least 10 participants;
- Partially edentulous patients receiving Implant Fixed Dental Prostheses (IFDPs);
- Implant placement and implant loading protocols were specifically reported;
- Implant success criteria were reported;
- Minimum follow‐up period of 1 year;
- Root‐form or cylindrical implant with a rough surface;
- Intra‐osseous implant diameter between 3 and 6 mm.

The exclusion criteria were the following:

- Animal or in vitro studies;
- Zirconia implants;
- Short implants (less than 6 mm);
- Implants with machined surfaces or hydroxyapatite (HA) coatings;
- Implants supporting full‐arch restorations or removable appliances;
- Implants placed in irradiated bone or alveolar clefts;
- Data retrieved from chart reviews or questionnaires;
- Insufficient information provided on implant placement protocol;
- Insufficient information provided on loading protocol or type of implant superstructures;
- Insufficient information provided to determine implant survival rate or success rate;
- Insufficient information provided to identify success criteria.

When multiple articles focused on the same study group, only the one with the longest follow‐up was used to report results. Earlier publications were consulted only for any details missing from the latest one.

Studies related to implant rehabilitation in both partially edentulous and fully edentulous patients would only be included when success/survival data were separated between these two different population groups.

### 2.4 Screening of Studies

Three reviewers (JFPC, SA, and KP) independently conducted the screening and data extraction. Any disagreements were addressed through discussion, and when necessary, resolved in consultation with an additional reviewer (GOG).

### 2.5 Data Collection

Information was extracted from the included studies using standardized forms and included details such as study design, timing of implant placement after extraction, timing of functional loading, average follow‐up duration, number of patients and implants, treatment location, implant specifications (e.g., diameter, length, type, and surface), flap design, use of bone grafts or surgical guides, methods of assessing implant stability, application of intention‐to‐treat (ITT) analysis, occlusal contact of the provisional restoration, final prosthesis configuration, defined success criteria, timing of any failures, as well as implant and prosthesis survival and success rates.

When clarification or additional data were required, study authors were contacted directly via email.

### 2.6 Quality Assessment

Three independent reviewers (JFPC; SA and KP) assessed the methodological quality of all included comparative studies. The assessment of randomized controlled trials (RCTs) focused on their bias risk by using the Cochrane quality assessment tool specifically designed for RCTs (ROB2) [28]. Meanwhile, the Newcastle–Ottawa scale (NOS) [29] was applied to appraise the quality of controlled clinical trials (CCTs).

### 2.7 Validation Criteria

To support clinical recommendations and draw conclusions across different placement and loading protocols, the included studies were evaluated based on their design, sample size, and consistency of outcomes (outcome homogeneity, OH). Outcome homogeneity was considered positive (OH+) if the variation in implant survival rates for a given protocol was 10% or less, and negative (OH−) if it exceeded 10%, as described by [1]. Based on these parameters, levels of scientific and/or clinical validation were defined as follows:

Scientifically and clinically validated (SCV):

- Systematic reviews of RCTs; or
- Two or more RCTs + ≥ 100 patients + OH+; or
- One RCT and two or more prospective studies + ≥ 150 patients + OH+

Clinically well‐documented (CWD):

- One RCT and two or more prospective studies + ≥ 40 patients + OH+; or
- No RCTs but at least three prospective studies + ≥ 60 patients + OH+; or
- No RCTs but two or fewer prospective studies + ≥ 100 patients + OH+

Clinically documented (CD):

- No RCTs, at least two prospective + any retrospective studies + ≤ 40 patients + OH−; or
- No RCTs, retrospective studies + ≥ 60 patients + OH−/+

Clinically insufficiently documented (CID):

- None of the above, expert opinion only, case report only.

### 2.8 Statistical Analysis

The inter‐reviewer agreement was evaluated using Cohen's kappa to assess consistency in study selection and data extraction. Descriptive statistics were employed to report the success and survival rates of different implant placement and loading protocols. However, due to heterogeneity in the definition of implant success among studies, success rates were summarized descriptively without quantitative synthesis. Implant survival rates were therefore used as the primary quantitative outcome measure for comparison across protocols.

A mean cumulative survival rate for each of the implant placement and loading protocols was calculated and weighted by the duration of patient follow‐up and number of implants. The weighted average of survival rate is calculated as follows:

$$\bar{x}=\frac{X_{1}t_{1}n_{1}+X_{2}t_{2}n_{2}+\ldots +X_{k}t_{k}n_{k}}{t_{1}n_{1}+t_{2}n_{2}+\ldots +t_{k}n_{k}}\times 100\%$$

*X* = survival rate reported in the included study; *t* = follow‐up period; *n* = number of implants. All studies included in this SR were carefully selected according to their described research variables. For each study, we looked for clear information on the placement and loading protocols to be one of the variables studied/reported. To ensure robustness and reliability, a systematic approach was used to identify and remove outlier studies that could disproportionately influence results. Outlier identification was performed using boxplots and standardized *z*‐scores (±3) to detect extreme survival values within each protocol. These objective, distribution‐based criteria were applied a priori to evaluate the influence of statistical extremes on the weighted cumulative survival rates. All studies remained included in the qualitative synthesis; studies identified as potential outliers were retained in the main analysis and excluded only in sensitivity analyses to test robustness.

## 3 Results

**FIGURE 1:**
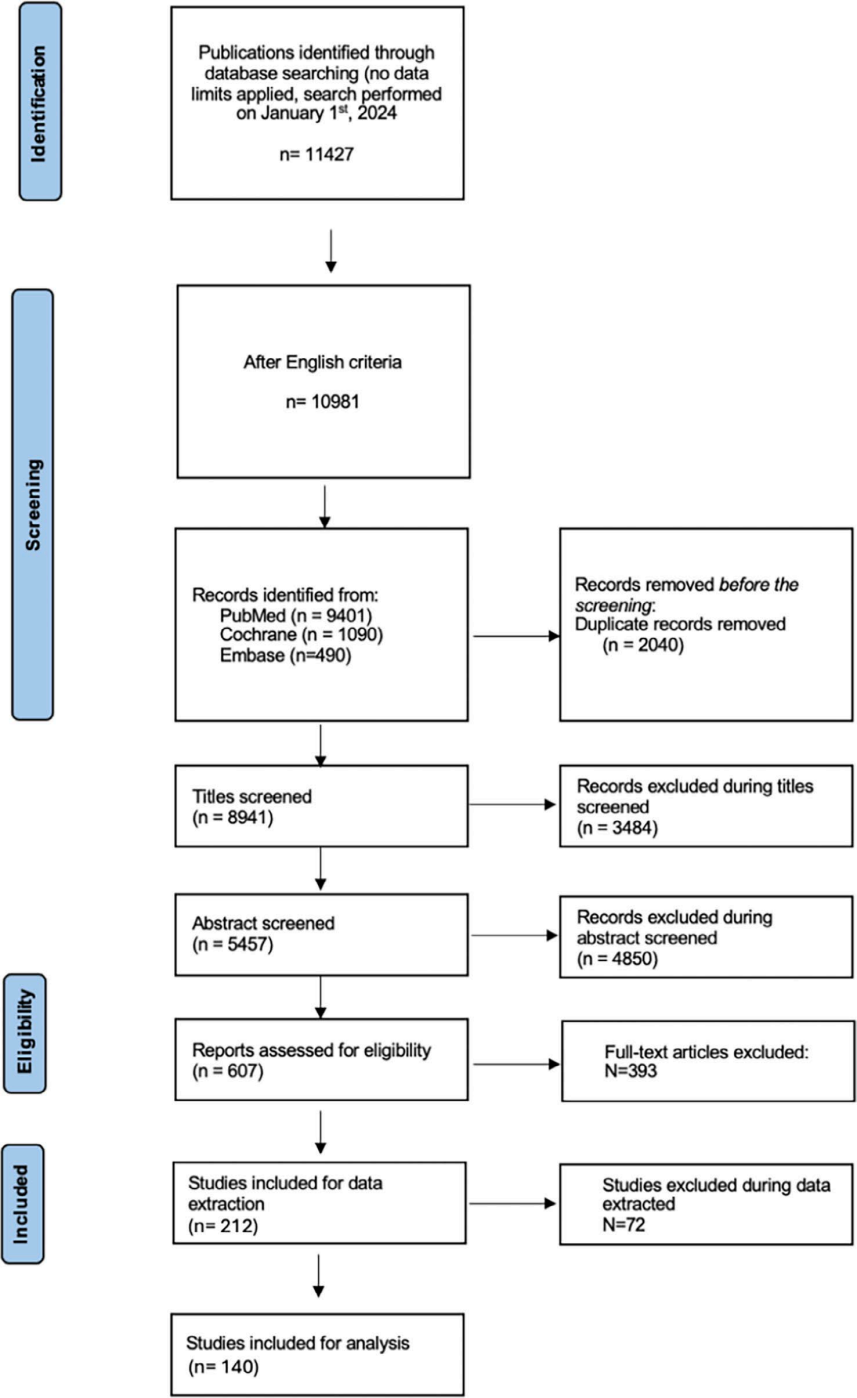
Search strategy.

The search retrieved 11 427 citations. Restricting the results to English left 10 981 records. After eliminating 2040 duplicates, 8941 unique titles were screened. Title filtering was followed by abstract appraisal of 5457 records, of which 4850 were excluded. Full‐text review of 607 articles removed a further 393. Data extraction initially included 214 studies, but 72 were subsequently excluded (Table 1). Consequently, 140 articles fulfilled all eligibility criteria and were incorporated into the qualitative synthesis (Figure 1). Among the 140 included studies, 42 were RCTs, 15 were CCTs and 83 were cohort studies. These collectively encompassed a total of 10 456 implants evaluated across various placement and loading protocols.

**TABLE 1:** Studies excluded during data extraction.

Reason for exclusion	Number	Studies
Insufficient information to separate partially and completely edentulous patients	6	Degidi et al. [30], Horwitz and Machtei [31], Malchiodi et al. [32, 33], Siebers et al. [34], Vandeweghe et al. [35]
Insufficient information to separate implant failure from partially and completely edentulous patients	6	Bekcioglu et al. [36], Danza, et al. [37], Glauser et al. [38], Kopp et al. [39], Penarrocha‐Diagoet al. [40], Barwacz et al. [41]
Less than 10 partially edentulous patients	4	Amato et al. [42], Polizzi and Cantoni [43], Mangano et al. [44], Donos et al. [45]
Insufficient follow‐up	3	Kotb et al. [46], Zhang et al. [47], Padmasree et al. [48]
Not screw‐type implant	2	Kopp et al. [39], Mangano et al. [44]
Short implants	5	Weerapong et al. [49], Guljé et al. [50], Anitua et al. [51], Cannizzaro et al. [52], Thoma et al. [53]
Intra‐osseous implant diameter more than 6.0 mm	3	Nedir et al. [54], Amato et al. [55], Atieh et al. [56]
Insufficient information to separate machined surface implants and rough surface implants	2	Wagenberg et al. [57]
Insufficient information of failed implants in different placement protocol	10	Arora et al. [58, 59], Daher et al. [60], Matar et al. [61], Zuiderveld et al. [62], Glauser et al. [63], Glauser [64], Ostman et al. [65], Daher et al. [66], Kim et al. [67]
Insufficient information of failed implants in different loading protocol	1	Wilson et al. [68]
Study scope focusing on grafting techniques	13	Nedir et al. [69], Meloni et al. [70], Mau et al. [71], Tsai et al. [72], Zhao et al. [73], Nedir et al. [69], Arora/Ivanovsi [58, 59], Merli [74], Alayan [75], Vincent [76], Lang et al. [77], Siormpas et al. [78], Urban et al. [79]
Data retrieved from chart reviews	7	Anitua et al. [80], Al Amri et al. [81], Bell and Bell [82], El‐Chaar [83], Harel et al. [84], Ormianer and Palti [85], Pozzi et al. [86]
Multiple studies on the same population	10	Cooper et al. [87], Abi‐Aad et al. [88], Buser, Bornstein et al. [89], Buser, Chappuis, Kuchler et al. [90], Buser et al. [91, 92], Kan et al. [93], Mangano et al. [94], Schropp et al. [95], Shibly et al. [96]
Total	72	

An 89.7% concordance was found among the three reviewers for title and abstract screening, with a kappa coefficient of 0.82 (SE = 0.07, 95% CI [0.68, 0.96]). For full‐text eligibility, a 94.6% concordance was achieved, with a kappa coefficient of 0.91 (SE = 0.05, 95% CI [0.81, 1.00]). The concordance strength was classified as “substantial” for title and abstract screening and “almost perfect” for full‐text selection. Any disagreements were resolved by consensus with a third reviewer.

Multiple publications based on the same patient cohort were consolidated into a single entry, prioritizing the most comprehensive and recent data available. When necessary, missing information was supplemented using earlier related studies.

While all included studies defined specific criteria for survival or success, the definitions varied significantly between studies, preventing the standardization of these criteria. Additionally, despite outlining success criteria, many studies only reported survival rates as the primary outcome measure. There was considerable heterogeneity in study designs, with a notable lack of RCTs and comparative studies that evaluated the same implant placement and loading protocol combinations. As a result, conducting a meta‐analysis of controlled studies was not feasible.

### 3.1 Quality Assessment for Including Comparative Studies

The RoB2 assessment was conducted on the 42 included RCTs to evaluate methodological quality. The majority of studies demonstrated appropriate randomization procedures (D1), with several reporting detailed methods for sequence generation and allocation concealment. However, blinding of participants and operators (performance bias, D2) was frequently lacking, leading to a high risk of bias across most trials.

In terms of missing outcome data (D3), several studies presented some concerns due to incomplete reporting, though attrition was generally low. Bias in measurement of the outcome (D4) was noted in multiple studies, particularly in trials where assessors were not blinded, increasing the likelihood of detection bias. Finally, bias in selection of the reported result (D5) was a concern in some cases, as certain studies selectively reported favorable outcomes.

Overall, while many studies exhibited low risk of bias in randomization and data completeness, the absence of blinding in several domains resulted in a moderate‐to‐high risk of bias in performance and detection measures. A detailed visualization of the RoB 2 assessments for each study is presented in Table 2.

**TABLE 2:** Risk of bias summary: Review authors' judgments about each risk of bias item for each included RCTs using the RoB2 assessment.

Study	D1	D2	D3	D4	D5	Overall
Hall et al. [97]	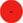	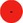	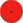	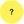	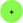	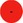
Ganeles et al. [98]	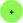	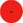	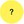	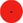	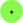	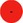
Schincaglia et al. [99]	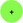	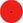	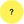	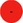	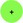	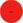
Shibly et al. [100]	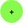	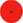	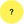	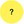	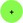	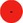
Van de Velde et al. [101]	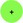	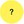	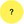	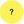	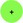	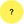
Margossian et al. [102]	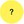	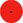	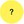	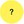	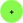	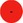
Schropp et al. [103]	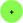	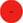	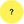	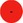	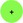	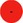
Felice et al. [104]	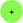	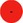	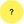	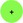	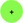	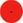
Kim et al. [67]	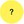	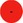	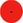	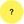	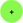	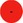
Gothberg et al. [105]	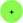	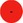	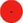	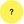	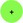	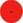
Malchiodi et al. [106]	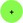	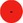	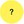	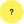	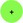	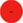
Slagter et al. [107]	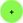	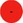	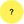	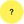	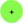	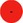
Bömicke et al. [108]	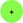	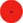	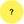	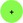	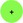	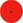
Cucchi et al. [109]	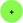	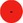	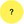	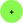	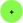	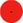
Gjelvold et al. [110]	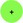	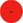	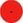	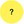	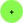	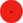
Esposito et al. [111]	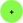	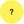	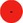	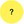	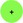	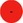
Donos et al. [112]	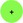	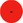	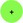	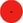	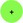	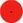
Mitsias et al. [113]	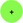	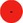	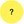	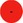	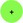	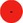
Raes et al. [114]	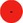	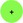	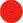	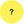	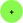	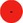
Meloni et al. [115]	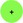	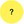	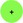	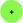	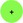	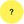
van Nimwegen et al. [116]	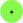	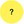	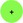	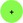	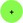	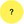
Khazaei et al. [117]	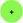	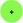	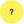	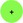	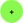	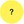
Daher et al. [66]	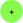	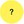	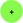	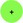	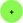	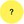
Nicolau et al. [118]	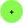	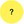	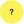	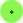	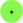	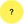
Rattanpanich et al. [119]	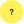	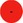	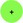	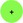	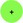	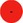
Merli et al. [120]	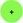	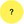	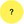	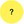	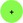	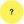
Lee et al. [121]	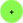	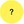	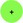	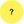	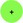	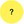
Felice et al. [122]	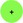	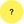	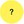	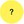	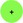	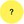
Slagter et al. [123]	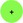	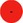	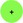	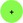	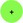	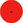
Wang et al. [124]	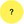	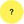	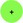	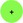	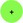	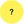
Wang et al. [125]	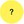	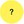	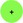	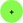	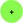	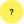
Slagter et al. [126]	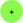	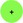	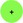	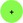	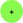	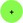
Linkevicius et al. [127]	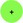	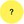	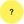	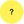	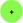	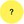
Gjevold et al. [110]	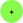	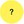	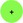	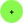	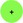	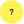
Galindo‐Morena et al. [128]	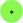	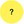	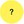	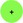	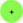	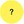
Testori et al. [129]	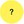	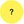	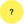	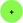	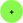	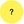
Albertini et al. [130]	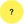	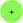	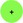	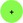	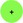	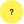
Puisys et al. [131]	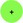	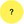	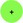	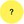	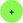	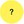
Kv et al. [132]	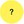	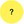	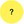	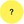	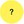	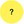
Grandi et al. [133]	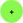	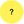	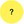	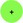	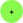	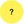
Strasding et al. [134]	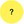	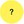	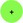	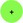	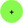	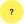
Fawzy et al. [135]	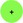	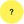	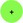	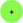	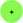	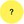

*Note:* Domains: D1: Risk of bias arising from the randomization process; D2: Risk of bias due to deviations from the intended interventions; D3: Missing outcome data; D4: Risk of bias in measurement outcome; D5: Risk of bias in selection. 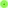, low risk; 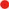, high risk; 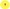, concerns.

For the CCTs, the NOS results of the 15 articles assessed are presented in Table 3. Most studies demonstrated strong cohort selection, with nearly all ensuring the representativeness of the exposed cohort and appropriately selecting the nonexposed cohort. Additionally, the ascertainment of exposure was well‐documented across all studies.

**TABLE 3:** Quality assessment and risk of bias of included CCTs using the NOS assessment.

Study	Representative of the exposed cohort	Selection of the nonexposed cohort	Ascertainment of exposure	Outcome of risk not present at commencement of study	Comparability of cases and controls	Assessment of outcome	Sufficient follow‐up time for outcomes to occur	Adequacy of follow‐up	Total
Achilli et al. [136]	*	*	*	*	*	*	*	*	8
Schropp and Isidor [137]	*	*	*	*	*	*	*	*	8
Mertens and Steveling [138]	*	*	*	*		*	*	*	7
De Bruyn et al. [139]	*	*	*	*	*	*	*	*	8
Heinemann et al. [140]	*	*	*	*	*	*	*		7
Vandeweghe et al. [141]	*	*	*	*		*	*		6
Meizi et al. [142]	*	*	*	*		*	*		6
Ormianer et al. [143]	*	*	*	*	*	*	*	*	8
Jokstad et al. [144]	*	*	*	*	*	*	*	*	8
Ayna et al. [145]	*	*	*	*		*	*		6
Salina et al. [146]	*	*	*	*	*	*	*		7
Menchini‐Fabris et al. [147]	*	*	*	*	*	*	*	*	8
Prati et al. [148]	*	*	*	*		*	*		6
Berberi et al. [149]	*	*	*	*		*	*	*	7
Amato et al. [150]	*	*	*	*	*	*	*		7

*Note:* An asterisk “*” indicates the study met the NOS criterion and received 1 point for that item (blank = 0). The Comparability domain can award up to 2 points; all other items award 1 point each. Maximum total = 9 points.

Regarding comparability, several studies adequately controlled for confounders, ensuring a moderate‐to‐high level of internal validity. However, some studies lacked sufficient adjustment for potential confounders, reducing their comparability scores.

In the outcome domain, most studies properly assessed the outcome of interest, although a few lacked sufficient follow‐up time or had concerns regarding adequate follow‐up rates. Overall, six studies achieved the highest NOS score of 8, indicating a low risk of bias, while others scored between 6 and 7, reflecting moderate methodological quality.

The predominance of studies with moderate‐to‐high risk of bias should be considered when interpreting the reported survival rates, as methodological limitations, heterogeneity, and incomplete blinding in individual studies may have influenced both outcome consistency and the overall strength of the evidence.

### 3.2 Outcome Analysis of Each Placement and Loading Protocol

The data extraction is outlined in Tables 4, 5, 6, 7, 8, 9, 10, 11, 12. The dataset was organized into 12 distinct treatment protocols (Table 13), according to the implant placement and loading classification system developed by [1]:

- Type 1A: immediate placement + immediate restoration/loading.
- Type 1B: immediate placement + early loading.
- Type 1C: immediate placement + conventional loading.
- Type 2A: early placement with soft tissue healing + immediate restoration/loading.
- Type 2B: early placement with soft tissue healing + early loading.
- Type 2C: early placement with soft tissue healing + conventional loading.
- Type 3A: early placement with partial bone healing + immediate restoration/loading.
- Type 3B: early placement with partial bone healing + early loading.
- Type 3C: early placement with partial bone healing + conventional loading.
- Type 4A: late placement + immediate restoration/loading.
- Type 4B: late placement + early loading.
- Type 4C: late placement + conventional loading.

**TABLE 4:** Data of the Type 1A protocol studies.

Study	Study design	Placement and loading protocol	Timing of placement	Timing of restoration/loading	Mean follow‐up (months)	No. of patients	No. of patients drop‐out	No. of implants	Implant type	Implant surface	No. of implant failed	Implant survival rate (%)	Implant success rate (%)	Prosthetic success rate (%)
Shibly et al. [100]	RCT	Type 1A	≤ 1 day	≤ 1 day	24	30	2	30	Nobel BL parallel	TiUnite	1	96.67	NR	NR
Felice et al. [104]^a^	RCT	Type 1A	≤ 1 day	≤ 1 day	12	16	0	16	Dentsply XiVE	NR	2	87.5	NR	100
Raes et al. [114]^a^	RCT	Type 1A	≤ 1 day	≤ 1 day	108	16	5	11	NR	Chemically modified	1	90.91	NR	NR
Merli et al. [120]	RCT	Type 1A	≤ 1 day	≤ 1 day	120	30	3	27	ELEMENT	Sand blasted acid‐etched surface	0	100	NR	NR
Wang et al. [124]^a^	RCT	Type 1A	≤ 1 day	≤ 1 day	12	20	2	18	Tapered, internal‐connection, 0.5 mm smooth collar	NR	2	88.89	NR	NR
Slagter et al. [126]	RCT	Type 1A	≤ 1 day	≤ 1 day	60	20	2	18	Nobel active	NR	0	100	NR	88.9
Puisys et al. [131]	RCT	Type 1A	≤ 1 day	≤ 1 day	12	25	0	25	Straumann BLT	SLA	0	100	100	100
Mertens and Steveling [138]	CCT	Type 1A	≤ 1 day	≤ 1 day	60	17	2	10	Dentsply	OsseoSpeed	0	100	100	100
De Bruyn et al. [139]	CCT	Type 1A	≤ 1 day	≤ 1 day	36	55	0	55	Dentsply	OsseoSpeed	3	94.55	87	NR
Vandeweghe et al. [141]	CCT	Type 1A	≤ 1 day	≤ 1 day	26	38	NR	23	Southern tapered	Moderately rough	0	100	NR	97.7
Meizi et al. [142]	CCT	Type 1A	≤ 1 day	≤ 3 days	12	155	NR	161	Saturn	NR	7	95.65	NR	NR
Menchini‐Fabris et al. [147]	CCT	Type 1A	< 1 day	< 1 day	36	28	NR	28	Outlink implants	Titanium plasma‐sprayed, rough surface	0	100	NR	NR
Berberi et al. [149]	CCT	Type 1A	< 1 day	< 1 day	120	22	4	22	Astra Tech TX implant system, Dentsply Implants	Rough	2	90.91	NR	NR
Amato et al. [150]	CCT	Type 1A	< 1 day	< 1 day	30	65	NR	77	Tapered internal or external hexagonal connection T3 implants (Zimmer Biomet)	Rough	2	97.4	NR	NR
De Rouck et al. [151]	Non‐comparative	Type 1A	≤ 1 day	≤ 1 day	12	30	1	30	Nobel BL tapered	TiUnite	1	96.67	NR	100
Blus and Szmukler‐Moncler [152]	Non‐comparative	Type 1A	≤ 1 day	≤ 1 day	12	23	NR	6	NR	NR	0	100	NR	NR
Valentini et al. [153]	Non‐comparative	Type 1A	< 1 week	< 1 week	33.6	40	NR	43	Dentsply	TiOblast	2	95.35	NR	NR
Becker et al. [154]	Non‐comparative	Type 1A	≤ 1 day	≤ 3 days	12	100	NR	100	Straumann TL parallel	SLActive	1	99	99	100
Brown and Payne [155]	Non‐comparative	Type 1A	≤ 1 day	≤ 1 day	12	25	0	26	Co‐axis TL tapered	Roughened surfaces	0	100	NR	92.31
Kan et al. [156]	Non‐comparative	Type 1A	≤ 1 day	≤ 1 day	48	35	0	35	Nobel BL tapered	HA	0	100	100	NR
Mura [157]	Non‐comparative	Type 1A	≤ 1 day	≤ 1 day	60	48	8	66	Nobel BL tapered	TiUnite	0	100	NR	98.5
Hartlev et al. [158]	Non‐comparative	Type 1A	≤ 1 day	≤ 1 day	33	55	13	55	Nobel BL tapered	TiUnite	1	98.18	NR	100
Mangano et al. [159]	Non‐comparative	Type 1A	≤ 1 day	≤ 1 day	31.09	22	0	22	NR	NR	0	100	100	100
Paul and Held [160]	Non‐comparative	Type 1A	≤ 1 day	≤ 1 day	40.8	26	2	31	Nobel	NR	0	100	100	NR
Malchiodi et al. [161]	Non‐comparative	Type 1A	≤ 1 day	≤ 1 day	36	58	0	64	NR	FBR	0	100	100	NR
Grandi et al. [162]	Non‐comparative	Type 1A	≤ 1 day	≤ 1 day	12	25	0	25	JDentalCare tapered	Dual acid etched	0	100	NR	100
Noelken et al. [163]	Non‐comparative	Type 1A	≤ 1 day	≤ 1 day	27	19	1	34	NR	OsseoSpeed	0	100	100	NR
Calvo‐Guirado et al. [164]	Non‐comparative	Type 1A	≤ 1 day	≤ 1 day	36	53	NR	71	MIS	Rough	0	100	NR	NR
Cristalli et al. [165]	Non‐comparative	Type 1A	≤ 1 day	≤ 1 day	12	24	0	25	Nobel BL tapered	TiUnite	2	92	91.67	NR
Migliorati et al. [166]	Non‐comparative	Type 1A	≤ 1 day	≤ 1 day	24	47	1	47	Straumann BL tapered	SLActive	0	100	NR	NR
Kolerman et al. [167]	Non‐comparative	Type 1A	≤ 1 day	≤ 1 day	29	34	NR	34	MIS BL	NR	0	100	88	NR
Van Nimwegen et al. [168]	Non‐comparative	Type 1A	≤ 1 day	≤ 1 day	48	51	NR	64	Biomet 3i	Osseotite	2	96.88	NR	NR
Noelken et al. [169]	Non‐comparative	Type 1A	1 day	1 day	68	37	2	33	OsseoSpeed implants (Dentsply Sirona Implants, Mannheim, Germany)		0	100	97	NR
van Nimwegen et al. [116]	Non‐comparative	Type 1A	≤ 1 day	≤ 1 day	12	30	5	25	NobelActive; Nobel Biocare AG, Gothenburg, Sweden	NR	1	96	NR	NR
Zuiderveld et al. [62]	Non‐comparative	Type 1A	≤ 1 day	≤ 1 day	12	60	0	60	NobelActive tapered	NR	2	96.67	96.70	NR
Velasco Ortega et al. [170]	Non‐comparative	Type 1A	< 1 day	< 1 day	48	56	0	116	IPX Galimplants	SLA	3	97.41	97.4	100
Bonnet et al. [171]	Non‐comparative	Type 1A	< 1 day	< 1 day	48	30	0	30	Nobel replace	Anodized and rough	0	100	NR	NR
Koutuzis et al. [172]	Non‐comparative	Type 1A	< 1 day	< 1 day	12	28	1	28	Ankylos implants (Dentsply Sirona Implants)	rough	1	96.43	NR	NR
Ferrantino et al. [173]	Non‐comparative	Type 1A	≤ 1 day	≤ 1 day	12	59	1	58	Imax, iRES SAGL	NR	2	96.55	94.9	NR
Azaripour et al. [174]	Non‐comparative	Type 1A	≤ 1 day	≤ 2 h	36	20	0	20	Semados microstructured shoulder rsx (bego)	NR	0	100	100	100
Carosi et al. [175]	Non‐comparative	Type 1A	1 day	1 day	12	40	0	40	Straumann BLX	Chemically modified sandblasted	0	100	100	100
Azaripour et al. [176]	Non‐comparative	Type 1A	≤ 1 day	≤ 1 day	12	20	0	20	Semados RSX, Bego		0	100	NR	NR
Venkatraman et al. [177]	Non‐comparative	Type 1A	≤ 1 day	Immediate provisional	12	22	0	22	Adin, Touareg S	NR	0	100	100	100
Menchini‐Fabris et al. [178]	Non‐comparative	Type 1A	< 1 day	< 1 day	12	20	0	20	Titanium plasma‐sprayed implants (Prama, Sweden and Martina)	Rough	0	100	100	NR
Todescan et al. [179]	Non‐comparative	Type 1A	< 1 day	< 1 day	12	26	0	26	Nobel Active	Anodized and rough	0	100	100	NR

Abbreviations: BL, bone level implant; NR, not reported; RBM, resorbable blast media; SLActive, hydrophilic and chemically active sandblasted, large grit, acid etched; TL, tissue level implant.

^a^ Flagged for sensitivity analysis.

**TABLE 5:** Data of the Type 1B protocol studies.

Study	Study design	Placement and loading protocol	Timing of placement	Timing of restoration/loading	Mean follow‐up (months)	No. of patients	No. of patients drop‐out	No. of implants	Implant type	Implant surface	No. of implant failed	Implant survival rate (%)	Implant success rate (%)	Prosthetic success rate (%)
Strasding et al. [134]	RCT	Type 1B	≤ 1 day	8 weeks	13.2	30	Total 6, no info per group	27	BLT Roxolid	SLActive	1	96.3	96.67	96.3
Mertens and Steveling [138]	CCT	Type 1B	≤ 1 day	9.56 weeks	60	NR	NR	3	Dentsply	OsseoSpeed	1	66.67	97.14	100
Boronat et al. [180]	Non‐comparative	Type 1B	≤ 1 day	8 weeks (max); 6 weeks (mand)	12	30	12	16	DEFCON TSA	Avantblast	1	93.75	93.75	NR
Blus and Szmukler‐Moncler [152]	Non‐comparative	Type 1B	≤ 1 day	1 week to 3 months	12	NR	NR	24	NR	NR	0	100	NR	NR

Abbreviations: BLT, bone level tapered; NR, not reported; SLActive, hydrophilic and chemically active sandblasted.

**TABLE 6:** Data of the Type 1C protocol studies.

Study	Study design	Placement and loading protocol	Timing of placement	Timing of restoration/loading	Mean follow‐up (months)	No. of patients	No. of patients drop‐out	No. of implants	Implant type	Implant surface	No. of implant failed	Implant survival rate (%)	Implant success rate (%)	Prosthetic success rate (%)
Shibly et al. [100]	RCT	Type 1C	≤ 1 day	3 months	24	30		30	Nobel BL parallel	TiUnite	2	93.33	NR	NR
Felice et al. [104]	RCT	Type 1C	≤ 1 day	4 months	12	9	0	9	Dentsply XiVE	NR	0	100	NR	100
Malchiodi et al. [106]	RCT	Type 1C	≤ 1 day	3 months	12	20	0	20	SybronPRO XRT parallel	RBM	0	100	100	NR
Slagter et al. [107]	RCT	Type 1C	≤ 1 day	3 months	12	20	0	20	NR	NR	0	100	NR	NR
Cucchi et al. [109]	RCT	Type 1C	≤ 1 day	3 months	24.4	48	3	49	BTK BL tapered	Dual acid etched	2	95.92	NR	100
Esposito et al. [111]	RCT	Type 1C	≤ 1 day	≥ 4 months	16	70	3	67	NR	NR	4	94.03	NR	NR
Khazaei et al. [117]	RCT	Type 1C	≤ 1 day	≥ 6 months	12	13	0	13	SICvantage max, Switzerland	NR	0	100	NR	NR
Merli et al. [120]	RCT	Type 1C	≤ 1 day	≤ 6 weeks	120	30	3	27	ELEMENT and CONTACT	Sand blasted acid‐etched surface	1	96.3	NR	NR
Lee et al. [121]	RCT	Type 1C	≤ 1 day	≥ 4 months	12	10	0	10	BLT, Roxolid, SLActive	SLActive	0	100	NR	NR
Felice et al. [122]	RCT	Type 1C	≤ 1 day	≥ 4 months	36	70	5	65	NR	NR	5	92.31	NR	NR
Wang et al. [125]	RCT	Type 1C	≤ 1 day	4 months	12	20	0	20	NR	NR	0	100	NR	NR
Slagter et al. [126]	RCT	Type 1C	≤ 1 day	3 months	60	20	3	17	NobelActive	NR	0	100	NR	88.2
Slagter et al. [126]	RCT	Type 1C	≤ 1 day	3 months	60	20	3	17	NobelActive	NR	0	100	82.4	NR
Heinemann et al. [140]	CCT	Type 1C	≤ 1 day	5–6 months	24.8	35	NR	83	Dentaurum BL	Rough ceramic	0	100	100	NR
Meizi et al. [142]	CCT	Type 1C	≤ 1 day	Max: 6 months; mand: 3 months	12			54	Saturn	NR	3	94.44	NR	NR
Ormianer et al. [143]	CCT	Type 1C	< 1 day	> 3 months	104.4	108	NR	705	SPI, DFI, Arrow, TSV, Maestro	NR	44	93.76	NR	NR
Menchini‐Fabris et al. [147]	CCT	Type 1C	< 1 day	> 2 months	36	26	NR	26	Outlink implants (Sweden and Martina, Padova, Italy)	Titanium plasma‐sprayed, rough surface	0	100	NR	NR
Prati et al. [148]	CCT	Type 1C	< 1 day	> 3 months	36	16	1	16	2‐piece PRAMA implant	Anodized smooth machined surface with zirconium oxide particle blasts	0	100	100	100
Amato et al. [150]	CCT	Type 1C	< 1 day	> 3 months	30	47	NR	53	Tapered internal or external hexagonal connection T3 implants (Zimmer Biomet)	Rough	0	100	NR	NR
Mayer et al. [181]	Non‐comparative	Type 1C	≤ 1 day	6 months (max); 4 months (mand)	45.9	2		4	Biomet 3i	Osseotite Dual acid etched	0	100	NR	NR
Prosper et al. [182]	Non‐comparative	Type 1C	≤ 1 day	4–6 months	48	83	0	111	NR	Sand blasted	3	97.3	97.3	NR
Bianchi and Sanfilippo [183]	Non‐comparative	Type 1C	≤ 1 day	3–4 months	108	116	3	116	Straumann TL parallel	TPS	0	100	100	NR
Del Fabbro et al. [184]	Non‐comparative	Type 1C	≤ 1 day	3–4 months	18.5	30	2	61	BTI Biotechnology Institute	Acid etched	1	98.36	98.4	100
Blus and Szmukler‐Moncler [152]	Non‐comparative	Type 1C	≤ 1 day	3–6 months	12			10	NR	NR	0	100	NR	NR
De Angelis et al. [185]	Non‐comparative	Type 1C	≤ 1 day	3–4 months	12	80	1	80	Biomet 3i BL tapered	Dual acid etched	7	91.25	NR	NR
Fugazzotto [186]	Non‐comparative	Type 1C	≤ 1 day	3–7 months	62	64	NR	128	NR	NR	0	100	98.2	NR
Covani et al. [187]	Non‐comparative	Type 1C	≤ 1 day	6 months	120	91	7	159	Sweden and Martina	SLA	13	91.82	91.8	98,7
Covani et al. [188]	Non‐comparative	Type 1C	≤ 1 day	4 months	60	47	NR	47	Sweden and Martina	NR	2	95.74	NR	NR
Montoya‐Salazar et al. [189]	Non‐comparative	Type 1C	≤ 1 day	4.5 months	36	NR	NR	36	MIS	NR	1	97.22	NR	NR
Peñarrocha‐Oltra et al. [190]^a^	Non‐comparative	Type 1C	> 4 months	≤ 1 day	36	20	6	14	NR	NR	2	85.71		
Chan et al. [191]	Non‐comparative	Type 1C	≤ 1 day	≥ 4 months	12	38	2	36	IS II active (Neobiotech)	Smooth Collar	2	94.44	100	100
Dimaira et al. [192]	Non‐comparative	Type 1C	< 1 day	> 2 months	61.8	14	0	16	Porous tantalum trabecular implants (Zimmer TM implants)	Trabecular tantalum surface	1	93.75	NR	NR
Shi et al. [193]	Non‐comparative	Type 1C	< 1 day	> 3 months	12	44	1	43	NobelActive and NobelReplace Conical Connection implants	TiUnite (plasma‐sprayed)	0	100	100	100
Garcia‐Sanchez et al. [194]	Non‐comparative	Type 1C	≤ 1 day	8–10 weeks	12	28	2	26	Tapered biomometic OCEAN avinent	NR	0	100	100	NR

Abbreviations: BL, bone level implant; NR, not reported; RBM, resorbable blast media; SLActive, hydrophilic and chemically active sandblasted, large grit, acid etched; TL, tissue level implant.

^a^ Flagged for sensitivity analysis.

**TABLE 7:** Data of the Type 2–3A protocol studies.

Study	Study design	Placement and loading protocol	Timing of placement	Timing of restoration/loading	Mean follow‐up (months)	No. of patients	No. of patients drop‐out	No. of implants	Implant type	Implant surface	No. of implant failed	Implant survival rate (%)	Implant success rate (%)	Prosthetic success rate (%)
Esposito et al. [195]	RCT	Type 2‐3A	≥ 3 months	≤ 1 week	36	20	1	34	T3 Certain Tapered Prevail (BNPTXX), zimmer biomet	Dual acid etched and blasted up to top of the neck	2	94.12	94.44	78.95
Esposito et al. [195]	RCT	Type 2‐3A	≥ 3 months	≤ 1 day	36	20	2	38	T3 Certain Tapered Prevail (BNPTXX), zimmer biomet	Dual acid etched and blasted up to top of the neck	0	100	97.37	94.44
Meloni et al. [115]	RCT	Type 2‐3A	≥ 2 months	≤ 1 day	60	20	0	20	Nobel Replace Tapered Groovy	NR	0	100	90%	90
Rattanapanich et al. [119]	RCT	Type 2‐3A	≥ 4 months	Immediate	12	25	0	25	NDI, PW Plus	NR	0	100	100	100
Wang et al. [124]^a^	RCT	Type 2‐3A	> 3 months	≤ 1 day	12	26	1	26	NobelParallel conical connection	NR	0	100	100	100
Gjelvold et al. [196]	RCT	Type 2‐3A	≥ 4 months	Immediate	60	25	1	24	BioHorizons Tapered Internal Implant	Laser‐Lok	1	95.83	91.7	100
Albertini et al. [130]	RCT	Type 2‐3A	≥ 4 months	≤ 1 week	12	9	0	17	Vega‐Cti (Klocker)	Thermo‐chemically treated, ContactTi surface	0	100	100	88.24 provisional phase, 100 definitive phase

Abbreviations: BLT, bone level tapered; NR, not reported; SLActive, hydrophilic and chemically active sandblasted.

^a^ Flagged for sensitivity analysis.

**TABLE 8:** Data of the Type 2–3B protocol studies.

Study	Study design	Placement and loading protocol	Timing of placement	Timing of restoration/loading	Mean follow‐up (mo)	No. of patients	No. of patients drop‐out	No. of implants	Implant type	Implant surface	No. of implant failed	Implant survival rate (%)	Implant success rate (%)	Prosthetic success rate (%)
Lee et al. [121]	RCT	Type 2–3B	8 weeks	≥ 2 months	12	10	0	10	BLT, Roxolid, SLActive	SLActive	0	100	NR	NR
Strasding et al. [134]	RCT	Type 2–3B	6–8 weeks	8 weeks	13.2	30	6	27	BLT Roxolid	SLActive	0	100	100	96.3
Belser et al. [197]	Non‐comparative	Type 2–3B	4–8 weeks	6–12 weeks	31.44	45	4	45	Straumann TL parallel	SLA	0	100	100	NR

Abbreviations: BLT, bone level tapered; NR, not reported; SLActive, hydrophilic and chemically active sandblasted.

**TABLE 9:** Data of the Type 2–3C protocol studies.

Study	Study design	Placement and loading protocol	Timing of placement	Timing of restoration/loading	Mean follow‐up (months)	No. of patients	No. of patients drop‐out	No. of implants	Implant type	Implant surface	No. of implant failed	Implant survival rate (%)	Implant success rate (%)	Prosthetic success rate (%)
Schropp et al. [103]	RCT	Type 2–3C	10 days	3 months	120	22	4	22	Biomet 3i parallel	Osseotite	2	90.91	NR	NR
Malchiodi et al. [106]	RCT	Type 2–3C	> 12 weeks	3 months	12	20	0	20	SybronPRO XRT parallel	RBM	0	100	100	NR
Esposito et al. [111]	RCT	Type 2–3C	6 weeks	≥ 4 months	16	70	5	65	NR	NR	4	93.85	NR	NR
Meloni et al. [115]	RCT	Type 2–3C	≥ 2 months	4–5 months	60	20	0	20	Nobel Replace Tapered Groovy	NS	0	100	90%	80
Felice et al. [122]	RCT	Type 2–3C	6 weeks	≥ 4 months	36	70	9	61	NR	NR	4	93.44	NR	NR
Wang et al. [124]^a^	RCT	Type 2–3C	> 3 months	3 months	12	25	2	23	NobelParallel conical connection	NR	0	100	100	100
Gjelvold et al. [196]	RCT	Type 2–3C	≥ 4 months	4 months	60	25	2	23	BioHorizons Tapered Internal Implant	Laser‐Lok	1	95.65	83.3	95.7
Albertini et al. [130]	RCT	Type 2–3C	≥ 4 months	4 weeks	12	12	2	16	Vega‐Cti (Klocker)	Thermo‐chemically treated, ContactTi surface	0	100	100	100 provisional phase, 81.25 definitive phase
Puisys et al. [131]	RCT	Type 2–3C	6 weeks	≥ 4 months	12	25	0	25	Straumann BLT	SLA	0	100	100	100
Schropp and Isidor [137]	CCT	Type 2–3C	10 days	4–5 months	60	23	2	23	Biomet 3i parallel	Osseotite	2	91.3	NR	95.24
Ormianer et al. [143]	CCT	Type 2–3C	6–8 weeks	> 3 months	104.4	36	NR	225	SPI, DFI, Arrow, TSV, Maestro	Excludes machined surfaces	18	92	NR	NR
Prati et al. [148]	CCT	Type 2–3C	8 weeks	> 3 months	36	20	1	20	2‐piece PRAMA implant	Anodized smooth machined surface with zirconium oxide particle blasts	0	100	100	100
Buser, Chappuis, Bornstein et al. [198], Buser, Chappuis, Kuchler et al. [90]	Non‐comparative	Type 2–3C	4–8 weeks	8–12 weeks	84	41	8	41	Straumann TL parallel and tapered	SLA	0	100	NR	NR
Chappuis et al. [10]	Non‐comparative	Type 2–3C	4–8 weeks	8–12 weeks	120	20	0	20	Straumann BL	SLActive	0	100	95	NR
Jonker et al. [199]	Non‐comparative	Type 2–3C	≥ 12 weeks	8 weeks	12	45	1	44	Straumann bone level	NR	0	100	100	NR
Bruyckere et al. [200]	Non‐comparative	Type 2–3C	≥ 3 months	3 months	12	42	0	42	NobelActive	NR	0	100	100	NR

Abbreviations: BL, bone level implant; BLT, bone level tapered; NR, not reported; RBM, resorbable blast media; SLActive, hydrophilic and chemically active sandblasted, large grit, acid etched; TL, tissue level implant.

^a^ Flagged for sensitivity analysis.

**TABLE 10:** Data of the Type 4A protocol studies.

Study	Study design	Placement and loading protocol	Timing of placement	Timing of restoration/loading	Mean follow‐up (months)	No. of patients	No. of patients drop‐out	No. of implants	Implant type	Implant surface	No. of implant failed	Implant survival rate (%)	Implant success rate (%)	Prosthetic success rate (%)
Hall et al. [97]	RCT	Type 4A	NR	≤ 1 day	12	14	0	14	Southern tapered	Roughened	1	92.86	NR	92.3
Ganeles et al. [98]	RCT	Type 4A	≥ 4 months	≤ 1 day	12	138	NR	197	Straumann TL parallel	SLActive	4	97.97	NR	NR
Schincaglia et al. [99]	RCT	Type 4A	≥ 4 months	≤ 1 day	12	15	0	15	Nobel	TiUnite	1	93.33	NR	NR
Van de Velde et al. [101]	RCT	Type 4A	≥ 4 months	≤ 1 day	18	13	1	36	Straumann TL tapered	SLA	1	97.22	72.2	100
Margossian et al. [102]	RCT	Type 4A	≥ 4 months	≤ 1 day	24	80	0	209	Biomet 3i	Osseotite	7	96.65	96.7	NR
Kim et al. [67]	RCT	Type 4A	≥ 6 months	≤ 1 day	12	21	0	22	Straumann TL parallel	SLActive	3	86.36	NR	NR
Göthberg et al. [105]	RCT	Type 4A	> 3 months	< 2 days	12	26	0	78	Nobel BL	TiUnite	4	94.87	NR	NR
Bömicke et al. [108]	RCT	Type 4A	> 6 weeks	≤ 1 day	36	19	0	19	Nobel BL tapered	TiUnite	1	94.74	NR	84.2
Gjelvold et al. [110]	RCT	Type 4A	≥ 4 months	≤ 1 day	12	25	0	25	BioHorizons tapered	NR	0	100	96	100
Donos et al. [112]	RCT	Type 4A	≥ 4 months	≤ 1 day	24	12	0	12	Bone level	Hydrophilic (SLActive)	0	100	NR	NR
Mitsias et al. [113]	RCT	Type 4A	≥ 4 months	≤ 2 days	12	18	0	18	NR	NR	0	100	NR	NR
Raes et al. [114]^a^	RCT	Type 4A	≥ 4 months	≤ 1 day	108	23	5	18	NR		0	100	NR	NR
Meloni et al. [115]	RCT	Type 4A	> 4 months	≥ 4 months	60	20	0	40	Nobel replace, Tapared Groovy, Nobel Biocare	Anodized surface	0	100	NR	NR
Meloni et al. [115]	RCT	Type 4A	> 4 months	< 1 day	60	20	NR	20	Nobel Replace Tapered Groovy, Nobel Biocare	Anodised surface	0	100	NR	NR
Daher et al. [66]	RCT	Type 4A	≥ 9 months	≤ 72 h	36	23	3	80	NobelActive	NR	4	95	95	75
Nicolau et al. [118]	RCT	Type 4A	≥ 4 months	≤ 1 day	120	26	NR	33	Straumann Standard	SLActive	1	96.97	98.2	96.8
Linkevicius et al. [127]	RCT	Type 4A	≥ 4 months	3–7 days	12	32	0	32	NR	NR	0	100	NR	NR
Testori et al. [129]	RCT	Type 4A	≥ 4 months	≤ 48 h	180	25	12	28	Full Osseotite Tapered Implant FNT, Zimmer Biomet 3i	NS	1	96.43	78	96
Grandi et al. [133]	RCT	Type 4A	≥ 4 months	≤ 1 day	144	40	25	81	Tapered implants (JDEvolution, J Dental Care) with internal hex connection	Sand blasted acid‐etched surface up to the neck	0	100	Report not detailed enough for implant level calculation	97.53
Kv [132]	RCT	Type 4A	< 1 day	< 1 day	12	14	NR	14	Touareg S, Adin Dental Implant Systems	Rough	0	100	100	100
Fawzy et al. [135]	RCT	Type 4A	≥ 4 months	≤ 48 h	12	10	0	10	IS II active, neobiotech	NS	1	90	NR	NR
Achilli et al. [136]	CCT	Type 4A	≥ 3 months	≤ 1 day	12	21	0	43	Nobel BL tapered	TiUnite	0	100	100	NR
Mertens and Steveling [138]	CCT	Type 4A	NR	≤ 1 day	60	NR	NR	4	NR	NR	0	100	100	100
De Bruyn et al. [139]	CCT	Type 4A	NR	≤ 1 day	36	58	0	58	Dentsply	OsseoSpeed	1	98.28	92	NR
Vandeweghe et al. [141]	CCT	Type 4A	NR	≤ 1 day	26		NR	20	Southern tapered	Moderately rough	0	100	NR	
Meizi et al. [142]	CCT	Type 4A	≥ 3 months	≤ 3 days	12	NR	NR	23	Saturn	NR	0	100	NR	NR
Ayna et al. [145]	CCT	Type 4A	≥ 6 months	≤ 1 day	60	48	NS	48	Internal‐hexed self‐tapping (LGI plus, Hi‐Te implant), 6 mm	Large grit, sand blasted and acid etched	3	93.75	93.75	93.75
Berberi et al. [149]	CCT	Type 4A	> 6 months	< 1 day	120	20	4	22	Astra Tech TX implant system, Dentsply Implants	Rough	0	100	NR	NR
Luongo et al. [201]	Non‐comparative	Type 4A	NR	≤ 1 day *n* = 10; 2–11 days *n* = 30	12	40	0	82	Straumann TL parallel	SLA	1	98.78	97.5	NR
Ostman et al. [202]	Non‐comparative	Type 4A	≥ 4 months	≤ 1 day	48	NR	0	180	Nobel	TiUnite	1	99.44	NR	NR
Siddiqui et al. [203]	Non‐comparative	Type 4A	> 6 months	≤ 1 day	12	44	NR	51	Zimmer tapered	Microtextured	1	98.04	98.04	NR
Calandriello and Tomatis [204]	Non‐comparative	Type 4A	≥ 4 months	≤ 1 day	60	33	NR	40	Nobel BL tapered	TiUnite	2	95	95	NR
Degidi et al. [205]	Non‐comparative	Type 4A	NR	≤ 1 day	36	24	0	48	Ankylos Dentsply	SLA	0	100	100	100
Fung et al. [206]	Non‐comparative	Type 4A	≥ 4 months	≤ 1 day	36	10	0	20	Nobel BL	ADZ	0	100	100	85
Oyama et al. [207]	Non‐comparative	Type 4A	≥ 2 months	≤ 1 day	12	13	NR	17	Dentsply Xives	Grit‐blasted thermal acid etched	0	100	100	NR
Mangano et al. [159]	Non‐comparative	Type 4A	≥ 6 months	≤ 1 day	34.4	18	0	18	NR	NR	0	100	100	100
Lang et al. [208]	Non‐comparative	Type 4A	NR	≤ 1 day	60	20	5	20	Zimmer tapered	NR	1	95	NR	NR
Fugl et al. [209]	Non‐comparative	Type 4A	≥ 2 months	≤ 1 day	12	91	6	93	NR	NR	1	98.92	97	NR
Minichetti et al. [210]	Non‐comparative	Type 4A	> 6 months	< 1 day	75.5	24	NR	33	Tapered one‐piece implants (Zimmer Biomet)	Rough surface	0	100	100	NR
Cooper et al. [211]^a^	Non‐comparative	Type 4A	< 1 day	< 1 day	60	141	17	88	OsseoSpeed TX (Dentsply Sirona), NobelSpeedy (Nobel Biocare), NanoTite Certain PREVAIL (Biomet 3i)	Rough (SLA and Plasma‐sprayed)	14	84.09	NR	NR

Abbreviations: BLT, bone level tapered; NR, not reported; SLActive, hydrophilic and chemically active sandblasted.

^a^ Flagged for sensitivity analysis.

**TABLE 11:** Data of the Type 4B protocol studies.

Study	Study design	Placement and loading protocol	Timing of placement	Timing of restoration/loading	Mean follow‐up (months)	No. of patients	No. of patients drop‐out	No. of implants	Implant type	Implant surface	No. of implant failed	Implant survival rate (%)	Implant success rate (%)	Prosthetic success rate (%)
Ganeles et al. [98]	RCT	Type 4B	≥ 4 months	28–34 days	12	128	NR	186	Straumann TL parallel	SLActive	6	96.77	NR	NR
Van de Velde et al. [101]	RCT	Type 4B	≥ 4 months	6 weeks	18	13		34	Straumann TL tapered	SLA	0	100	82.35	100
Mitsias et al. [113]	RCT	Type 4B	≥ 4 months	6 weeks	12	18	0	18	NR	NR	0	100	NR	NR
Nicolau et al. [118]	RCT	Type 4B	≥ 4 months	28–34 days	120	30	NR	39	Straumann Standard	SLActive	2	94.87	97.1	NR
Linkevicius et al. [127]	RCT	Type 4B	≥ 6 months	2–3 months	12	32	5	31	NR	NR	0	100	NR	NR
Galindo‐Moreno et al. [128]	RCT	Type 4B	≥ 4 months	4 weeks	13	18	6	12	MIS	Multi‐phosphate coating	1	91.67	84.62	NR
	RCT	Type 4B	≥ 4 months	8 weeks	13	16	4	12	MIS	Multi‐phosphate coating	1	91.67	76.92	NR
Testori et al. [129]	RCT	Type 4B	≥ 4 months	2 months	180	27	11	30	Full Osseotite Tapered Implant FNT, Zimmer Biomet 3i	Microtextured surface	0	100	96.3	98.15
Grandi et al. [133]	RCT	Type 4B	≥ 4 months	2 months	144	40	11	80	Tapered implants (JDEvolution, J Dental Care) with internal hex connection	Sand blasted acid‐etched surface up to the neck	2	97.5	Report not detailed enough for implant level calculation	96.25
Achilli et al. [136]	CCT	Type 4B	≥ 3 months	6 weeks	12	33	0	69	Nobel BL tapered	TiUnite	0	100	100	NR
Mertens and Steveling [138]	CCT	Type 4B	NR	9.56 weeks	60			32	Dentsply	OsseoSpeed	NR	NR		100
Boronat et al. [180]	Non‐comparative	Type 4B	NR	8 weeks (max); 6 weeks (mand)	12	NR	NR	90	DEFCON TSA	Avantblast	2	97.78	97.78	NR
Bornstein et al. [212]	Non‐comparative	Type 4B	≥ 4 months	3 weeks	36	39	0	56	Straumann TL parallel	SLActive	0	100	100	NR
Karabuda et al. [213]	Non‐comparative	Type 4B	≥ 3 months	12 weeks (max); 8 weeks (mand)	15	22	0	96	Straumann TL parallel	SLA and SLA active	1	98.96	98.96	NR
Akca et al. [214]	Non‐comparative	Type 4B	NR	5–6 weeks	14	22	0	52	Straumann BL parallel	SLA	0	100	100	100
Maiorana et al. [215]	Non‐comparative	Type 4B	≥ 4 months	6–10 weeks	36	69		97	OsseoSpeed TX 3.0S, DENTSPLY Implants	NR	4	95.88	NR	NR
Sener‐Yamaner et al. [216]	Non‐comparative	Type 4B	≥ 4 months	3–8 weeks	81	55	NR	175	Straumann TL	SLA *n* = 48; SLActive *n* = 48	3	98.29	NR	NR
Gulje et al. [50]	Non‐comparative	Type 4B	≥ 4 months	6 weeks (splinted provisionals)	60	95	10	183	OsseoSpeed, Dentsply	NR	5	97.27	44.06 for 6 mm, 60.46 for 11 mm	NR
Levine et al. [217]	Non‐comparative	Type 4B	> 6 months	3–4 weeks	114.4	22	1	18	Strauman SLActive	Modified sandblasted and SLA	0	100	100	100
Salem et al. [218]	Non‐comparative	Type 4B	≥ 4 months	6 weeks	24	30	0	30	V Plus, Vitronex Elite Implant	NR	0	100	100	90 for two groups (C, P), 100 for one group (Z)

Abbreviations: BL, bone level implant; NR, not reported; RBM, resorbable blast media; SLActive, hydrophilic and chemically active sandblasted, large grit, acid etched; TL, tissue level implant.

**TABLE 12:** Data of the Type 4C protocol studies.

Study	Study design	Placement and loading protocol	Timing of placement	Timing of restoration/loading	Mean follow‐up (months)	No. of patients	No. of patients drop‐out	No. of implants	Implant type	Implant surface	No. of implant failed	Implant survival rate (%)	Implant success rate (%)	Prosthetic success rate (%)
Hall et al. [97]	RCT	Type 4C	NR	6 months	12	14	2	14	Southern tapered	Roughened	0	100	NR	85.7
Schincaglia et al. [99]	RCT	Type 4C	≥ 4 months	3–4 months	12	15	0	15	Nobel BL parallel	TiUnite	0	100	NR	NR
Margossian et al. [102]	RCT	Type 4C	≥ 4 months	NR	24	37	0	98	SybronPRO XRT parallel	RBM	0	100	100	NR
Schropp et al. [103]	RCT	Type 4C	> 3 months	3 months	120	22	1	22	Biomet 3i parallel	Osseotite	1	95.45	NR	NR
Schropp et al. [103]	RCT	Type 4C	17 months	3 months	120	19	2	19	Biomet 3i parallel	Osseotite	0	100	NR	NR
Felice et al. [104]^a^	RCT	Type 4C	4 months	4 months	12	19		19	Dentsply XiVE	NR	0	100	NR	100
Kim et al. [67]	RCT	Type 4C	≥ 6 months	20–23 weeks	12		0	24	Straumann TL parallel	SLActive	0	100	NR	NR
Göthberg et al. [105]	RCT	Type 4C	> 3 months	3 months	12	24	0	72	Nobel BL	TiUnite	2	97.22	NR	NR
Slagter et al. [107]	RCT	Type 4C	> 3 months	3 months	12	20	0	20	NR	NR	0	100	NR	NR
Bömicke et al. [108]	RCT	Type 4C	> 6 weeks	3 months	36	19	3	16	Nobel BL tapered	TiUnite	0	100	NR	68.8
Cucchi et al. [109]	RCT	Type 4C	> 3 months	3 months	24.4	44	4	48	BTK BL tapered	Dual acid etched	0	100	NR	100
Gjelvold et al. [110]	RCT	Type 4C	≥ 4 months	≥ 4 months	12	25	0	25	BioHorizons tapered	NR	1	96	88	100
Esposito et al. [111]	RCT	Type 4C	≥ 4 months	≥ 4 months	16	70	6	64	NR	NR	1	98.44	NR	NR
Donos et al. [112]	RCT	Type 4C	≥ 4 months	4 months	24	12	0	12	Bone Level implant	Hydrophilic (SLActive)	0	100	NR	NR
Mitsias et al. [113]	RCT	Type 4C	≥ 4 months	3 months	12	18	2	16	NR	NR	0	100	NR	NR
Meloni et al. [115]	RCT	Type 4C	> 4 months	≥ 4 months	60	30	5	25	Nobel replace, Tapared Groovy, Nobel Biocare	Anodized surface	1	96	NR	NR
Meloni et al. [115]	RCT	Type 4C	> 4 months	> 4 months	60	20	NR	20	Nobel Replace Tapered Groovy, Nobel Biocare	Anodised surface	0	100	NR	NR
Khazaei et al. [117]	RCT	Type 4C	≥ 4 months	≥ 6 months	12	13	0	13	SICvantage max, Switzerland	NR	0	100	NR	NR
Daher et al. [66]	RCT	Type 4C	≥ 9 months	≥ 3 months	36	23	3	80	NobelActive	NR	3	96.25	93.75	91.67
Rattanapanich et al. [119]	RCT	Type 4C	≥ 4 months	Delayed	12	25	0	25	NDI, PW Plus	NS	0	100	100	100
Wang et al. [125]	RCT	Type 4C	≥ 4 months	≥ 4 months	12	20	0	20	Tapered, internal‐connection, 0.5 mm smooth collar	NR	0	100	NR	NR
Felice et al. [122]	RCT	Type 4C	≥ 4 months	≥ 4 months	36	70	8	62	NR	NR	1	98.39	NR	NR
Kv [132]	RCT	Type 4C	< 1 day	> 3 months	12	14	NR	14	Touareg S, Adin Dental Implant Systems	Rough	0	100	100	100
Fawzy et al. [135]	RCT	Type 4C	≥ 4 months	3 months	12	10	0	10	IS II active, neobiotech	NS	1	90	NR	NR
Schropp and Isidor [137]	CCT	Type 4C	> 3 months	4–5 months	60	22		22	Biomet 3i parallel	Osseotite	1	95.45	NR	
Heinemann et al. [140]	CCT	Type 4C	≥ 6 months	5–6 months		23	NR	53	Tapered	Blasted	0	100	100	NR
Meizi et al. [142]	CCT	Type 4C	≥ 3 months	Max: 6 months; mand: 3 months	12			106	Saturn	NR			NR	NR
Ormianer et al. [143]	CCT	Type 4C	4–6 months	> 3 months	104.4	25	NR	182	SPI, DFI, Arrow, TSV, Maestro	Excludes machined surfaces	5	97.25	NR	NR
Jokstad et al. [144]	CCT	Type 4C	> 6 months	> 3 months	12	20	0	41	Osseospeed TX, Replace Select Ti‐Unite, Straumann Bone Level SLActive	SLActive and Ti‐Unite surfaces	0	100	100	100
Ayna et al. [145]	CCT	Type 4C	≥ 6 months	3 months	60	15	NS	15	Internal‐hexed self‐tapping (LGI plus, Hi‐Te implant)	Large grit, sandblased and acid‐etched	0	100	100	100
Salina et al. [146]	CCT	Type 4C	> 6 months	> 3 months	36	60	1	120	Tapered implant Axiom REG (Anthogyr, Sallanches, France) with internal morse tapered connection and platform switching	Airborne‐particle‐abraded with biphasic calcium phosphate (BCP) bioceramics	2	98.33	NR	
Prati et al. [148]	CCT	Type 4C	> 6 months	> 3 months	36	20	1	30	2‐piece Prama implant	Anodized smooth machined surface with zirconium oxide particle blasts	0	100	100	
Mayer et al. [181]	Non‐comparative	Type 4C	NR	6 months (max); 4 months (mand)	45.9	57	2 implants	67	Biomet 3i	Osseotite Dual acid etched	1	98.51	98.51	NR
Romeo et al. [219]	Non‐comparative	Type 4C	> 6 months	3–6 months	84	109	6	187	Straumann TL parallel	TPS	9	95.19	93.6	NR
Barone et al. [220]	Non‐comparative	Type 4C	≥ 3 months	3 months	12	116	0	112	Blossom BL tapered	NR	3	97.32	93.1	NR
Meloni et al. [221]	Non‐comparative	Type 4C	NR	3 months	36	18	0	36	Nobel BL tapered	TiUnite	0	100	NR	100
Esposito et al. [195]	Non‐comparative	Type 4C	> 4 months	> 4 months	12	30	2	28	NR	NR	2	92.86	NR	NR
Spinato et al. [222]	Non‐comparative	Type 4C	≥ 4 months	≥ 4 months	18	80	3	74	Shape1BC, I‐RES		3	95.95	NR	NR
Esposito et al. [223]	Non‐comparative	Type 4C	≥ 5 months	3 months	60	60	2	120	Tapered implant axiom REG (Anthogyr)	Airborne‐particle‐abraded with biphasic calcium phosphate (BCP) bioceramics consisting of a mixture of hydroapatite and beta‐TCP and then mild acid treatment	4	96.67	93.1	98.28
Waller et al. [224]	Non‐comparative	Type 4C	> 6 months	Late (6 months)	90	22	2	26	Camlog Screw line	NR	0	100	NR	NR
Kahramonoglu et al. [225]	Non‐comparative	Type 4C	> 6 months	> 2 months	60	30	6	62	Straumann SLActive, Astra OsseoSpeed, Thommen SPI	Sandblasted and acid‐etched surfaces	0	100	100	100
Meloni et al. [226]	Non‐comparative	Type 4C	> 6 months	> 3 months	60	18	1	34	NobelReplace Tapered Groovy implants (Nobel Biocare)	Anodised rough surface	0	100	100	100
Spinato et al. [222]	Non‐comparative	Type 4C	> 6 months	> 3 months	18	80	3	80	Shape1BC, I‐RES implants	Platform‐switched internal hex implants with rough surface	3	96.25	NR	NR
Esposito et al. [227]	Non‐comparative	Type 4C	≥ 3 months after extraction or ≥ 6 months after augmentation	3–4 months	12	31	8	62	Ticare inhex implants (Mozo‐grau)	RBM (Resorbable blast media) titanium surface	2	96.77	97.83	97.83
Siegenthaler et al. [228]	Non‐comparative	Type 4C	3–4 months	3–4 months	12	47	3	44	OsseoSpeed EV Astra Tech	NR	0	100	NR	NR
Meijndert et al. [229]	Non‐comparative	Type 4C	> 6 months	> 3 months	12	30	0	30	Straumann BLT	SLActive	0	100	100	NR
Stacchi et al. [230]	Non‐comparative	Type 4C	≥ 4 months	≥ 4 months	12	55	4	102	AnyRidge, MegaGen, Gyeongbuk, South Korea	Fully sandblasted, large grit, acid‐etched with calcium ions incorporated (Xpeed, MegaGen)	0	100	NR	NR

Abbreviations: BL, bone level implant; NR, not reported; RBM, resorbable blast media; SLActive, hydrophilic and chemically active sandblasted, large grit, acid etched; TL, tissue level implant.

^a^ Flagged for sensitivity analysis.

**TABLE 13:** Classification according to the implant placement and loading protocol.

	Loading protocol														
	Immediate restoration/loading (Type A)					Early loading (Type B)					Conventional loading (Type C)				
	Type	Weighted mean survival (%)	Mean follow‐up (months)	No of included implants	No of studies	Type	Weighted mean survival (%)	Mean follow‐up (months)	No. of included implants	No. of studies	Type	Weighted mean survival (%)	Mean follow‐up (months)	No. of included implants	No. of studies
Implant placement protocol															
Immediate placement (Type 1)	1A	98.01 (92–100)	32.9 (12–120)	1859	16^a^ 32^b^	1B	91.62 (66.67–96.3)	24.3 (12–60)	70	2^a^ 2^b^	1C	95.04 (92.31–100)	38.6 (12–120)	2188	19^a^ 15^b^
Early placement (Type 2–3)	2‐3A	97.82 (94.12–100)	32.6 (12–60)	184	7^a^ 0^b^	2‐3B	100.00	18.9 (12–31.44)	82	2^a^ 1^b^	2‐3C	94.02 (91.3–100)	48 (12–120)	690	12^a^ 4^b^
Conventional placement (Type 4)	4A	97.17 (93.33–100)	42.1 (12–180)	1915	29^a^ 12^b^	4B	97.85 (91.67–100)	48.9 (12–180)	1308	10^a^ 10^b^	4C	97.54 (95.45–100)	34.6 (12–36)	2160	31^a^ 15^b^

*Note:* Range of results indicated in brackets. Type 1A: immediate placement + immediate restoration/loading; Type 1B: immediate placement + early loading; Type 1C: immediate placement + conventional loading; Type 2–3A: early placement + immediate restoration/loading; Type 2–3B: early placement + early loading; Type 2–3C: early placement + conventional loading; Type 4A: late placement + immediate restoration/loading; Type 4B: late placement + early loading; Type 4C: late placement + conventional loading. CD (yellow): clinically documented; CWD (green): clinically well documented; SCV: scientifically and clinically validated.

^a^ No. of comparative studies.

^b^ No. of non‐comparative studies.

Due to the lack of clear distinctions in the timing of implant placement in many clinical study reports, implants following early loading protocols (Types 2 and 3) were grouped for this review.

From each study, the number of participants intended to receive the specified placement/loading protocol and the number who actually commenced that protocol were abstracted as reported by the authors. An ITT adherence metric was calculated for each study as ITT% = (N_treated/N_intended) × 100. Studies explicitly reporting no pretreatment exclusions were recorded as 100% ITT. When pretreatment exclusions were described (e.g., insufficient primary stability, inadequate bone volume/need for grafting, buccal bone dehiscence/fenestration, protocol deviations, or loss to follow‐up prior to treatment), ITT% was computed from the reported counts; if data were insufficient to derive ITT, the study was coded as not reported. Data extraction and ITT calculations were performed independently by two reviewers, with disagreements resolved by a third reviewer. ITT% was used descriptively to indicate potential selection bias at allocation and to contextualize survival estimates.

#### 3.2.1 Type 1A—Immediate Placement + Immediate Restoration/Loading

Sixteen comparative studies and 32 non‐comparative studies provided data on a total of 1859 implants placed using the Type 1A protocol. The weighted cumulative survival rate was 98.01% (range: 92%–100%) with a mean follow‐up of 32.9 months (range: 12–120 months). Success rates ranged between 92% and 100%. The individual characteristics and outcomes of studies reporting the Type 1A protocol are summarized in Table 4, which detail sample sizes, implant locations, follow‐up periods, and survival data for each included study.

#### 3.2.2 Type 1B—Immediate Placement + Early/Loading

Two clinical trials and two non‐comparative studies provided data on the outcomes of implants following the Type 1B protocol. Of the 70 Type 1B implants, 6 failed. The weighted cumulative survival rate was 91.62% (range: 66.67%–96.3%) with a mean follow‐up of 24.3 months (range: 12–60 months). Success rates ranged between 66.67% and 96.3%. The studies evaluating the Type 1B protocol are presented in Table 5, providing detailed information for each included study.

#### 3.2.3 Type 1C Immediate Placement + Conventional/Loading

Nineteen comparative studies and 16 non‐comparative studies provided data on the outcomes of implants following the Type 1C protocol. Of the 2188 implants, 108 failed. The weighted cumulative survival rate was 95.04% (range: 92.31%–100%) with a mean follow‐up of 38.6 months (range: 12–120 months). Success rates ranged between 92.31% and 100%. The studies corresponding to the Type 1C protocol are listed in Table 6.

#### 3.2.4 Type 2–3A—Early Placement + Immediate Restoration/Loading

Seven clinical trials provided data on the outcomes of implants following the Type 2–3A protocol. Of the 184 implants included, there were four failures. The weighted cumulative survival rate was 97.82% (range: 94.12%–100%) with a mean follow‐up of 32.6 months (range: 12–60 months). Success rates ranged between 94.12% and 100%. The included studies assessing the Type 2–3A protocol are summarized in Table 7.

#### 3.2.5 Type 2–3B—Early Placement + Early Loading

Two clinical trials and one non‐comparative study provided data on the outcomes of implants following the Type 2–3B protocol. Of the 82 implants included, none failed. The weighted cumulative survival rate was 100% with a mean follow‐up of 18.9 months (range: 12–31.44 months). The characteristics and outcomes of studies investigating the Type 2–3B protocol are presented in Table 8.

#### 3.2.6 Type 2–3C—Early Placement + Conventional/Loading

Twelve clinical trials and four non‐comparative studies provided data on the outcomes of implants following the Type 2–3C protocol. Of the 690 implants included, 42 failed. The weighted cumulative survival rate was 94.02% (range: 91.3%–100%) with a mean follow‐up of 48 months (range: 12–120 months). Success rates ranged between 91.3% and 100%. The studies included under the Type 2–3C protocol are summarized in Table 9.

#### 3.2.7 Type 4A—Late Placement + Immediate Restoration/Loading

Twenty‐nine comparative studies and 12 non‐comparative studies provided data on the outcomes of implants following the Type 4A protocol. Of the 1915 implants included, 54 failed. The weighted cumulative survival rate was 97.17% (range: 93.33%–100%) with a mean follow‐up of 42.1 months (range: 12–180 months). Studies following the Type 4A protocol are reported in Table 10.

#### 3.2.8 Type 4B—Late Placement + Early Loading

Ten clinical trials and nine non‐comparative studies provided data on the outcomes of implants following the Type 4B protocol. Of the 1308 implants included, 28 failed. The weighted cumulative survival rate was 97.85% (range: 91.67%–100%) with a mean follow‐up of 48.9 months (range: 12–180 months). Success rates ranged between 91.67% and 100%. The data corresponding to the Type 4B protocol are listed in Table 11.

#### 3.2.9 Type 4C—Late Placement + Conventional Loading

Thirty‐one comparative studies and 15 non‐comparative studies provided data on the outcomes of implants following the Type 4C protocol. Of the 2160 implants included, 53 failed. The weighted cumulative survival rate was 97.54% (range: 95.45%–100%) with a mean follow‐up of 34.6 months (range: 12–120 months). Success rates ranged between 95.45% and 100%. The individual studies reporting outcomes for the Type 4C protocol are summarized in Table 12.

##### 3.2.9.1 Outliers' Treatment

Five studies were classified as statistical outliers [104, 114, 124, 190, 211] and were evaluated in prespecified sensitivity analyses. These studies reported survival rates of 87.5%, 86.0%, 90.9%, 100%, and 84.1%, with follow‐ups of 36, 36, 96–120, 12, and 36 months, respectively. Excluding them produced only minimal changes in the pooled weighted survival estimates.

### 3.3 Criteria for Implant Placement and Loading Protocol

Table 14 showed the criteria for selection of specific placement/loading protocols. These were generally presented separately for placement and loading protocols as follows:

**TABLE 14:** Criteria for placement and loading protocol and intention‐to‐treat analysis.

Study	Placement and loading protocol	Criteria for placement protocol	Criteria for loading protocol (immediate or early loading)	No. of patients intended to treat	No. of patients failed to treat	ITT (%)	Reason for exclusion
RCT							
Hall et al. [97]	Type 4A vs. Type 4C	No need for bone grafting or ridge augmentation	Primary implant stability could be achieved following placement	28	0	100	NR
Ganeles et al. [98]	Type 4A vs. Type 4B	Adequate bone quality and quantity	NR	NR	NR	NR	NR
Schincaglia et al. [99]	Type 4A vs. Type 4C	Adequate bone to place a 5 × 8.5 mm or larger implant	IT ≥ 20 N cm	NR	NR	NR	NR
Shibly et al. [100]	Type 1A vs. Type 1C	Extraction sockets with an open defect, lacking ≥ 1 bone walls	IT ≥ 35 N cm	72	12	83.3	Immediate implant placement not possible
Van de Velde et al. [101]	Type 4A vs. Type 4B	Adequate bone to place 2–3 4.1 × 8–12 mm implants	NR	14	2	85.7	Bone grafting required for implant placement; patient passed away
Margossian et al. [102]	Type 4A vs. Type 4C	Adequate bone height to place a 10 mm or longer implant	IT ≥ 30 N cm ISQ ≥ 60	117	0	100	NR
Schropp et al. [103]	Type 2–3C vs. Type 4C	NR	NR	72 suitable for single implant therapy; five patients withdrew during the period from tooth extraction to commencement of implant treatment	9	87.5	NR
Felice et al. [104]	Type 1A vs. Type 1C vs. Type 4A vs. Type 4C	< 4 mm of the buccal wall missing after tooth extraction	IT ≥ 35 N cm	55	5	90.9	Buccal bone dehiscence
Kim et al. [67]	Type 4A vs. Type 4C	Adequate bone to place 4.1/4.8 × 10/12 mm implants without bone augmentation procedures; Attached gingiva ≥ 2 mm	NR	21	0	100	NR
Gothberg et al. [105]	Type 4A vs. Type 4C	NR	Primary implant stability with ≥ 1 mm coverage of surrounding bone	NR	NR	NR	NR
Malchiodi et al. [106]	Type 1C vs. Type 4C	Extraction socket with a containing alveolus (4 bone‐wall defect); Bone height ≥ 9 mm in the maxilla and ≥ 11 mm in the mandible; Apical bone ≥ 3 mm	NR	40	0	100	NR
Slagter et al. [107]	Type 1C vs. Type 4C	Labial bony defect of ≥ 5 mm after tooth removal; sufficient bone on the palatal side	NR	40	0	100	NR
Bömicke et al. [108]	Type 4A vs. Type 4C	Bone height ≥ 12 mm, bone width ≥ 6 mm; Implant placement without grafting; Attached gingiva ≥ 4 mm	IT ≥ 35 N cm	38	0	100	NR
Cucchi et al. [109]	Type 1C vs. Type 4C	Adequate bone to place a 3.7 × 10 mm or larger implant without bone augmentation procedures	NR	102	3	97.1	Patient withdrawal
Gjelvold et al. [110]	Type 4A vs. Type 4C	No need for bone grafting or ridge augmentation	IT ≥ 30 N cm	62	12	80.6	Patient withdrawal; bone grafting required for implant placement
Esposito et al. [111]	Type 1C vs. Type 2–3C vs. Type 4C	Adequate bone to place a single implant at least 8.5 mm long with a minimal diameter of 3.5 mm	IT > 25 N cm	NR	NR	NR	NR
Donos et al. [112]	Type 4A vs. Type 4C	NR	IT ≥ 30 N cm	24	0	100	NR
Mitsias et al. [113]	Type 4A vs. Type 4B vs. Type 4C	Adequate bone to place one or more implants with minimal dimensions of 7.0 mm × 3.5 mm	IT ≥ 40 N cm	52	0	100	NR
Meloni et al. [115]	Type 4A vs. Type C Type 2–3A vs. Type 2–3C	Bone height ≥ 10 mm, bone width ≥ 6 mm; Keratinized gingiva ≥ 5 mm Bone height ≥ 10 mm, bone width ≥ 6 mm; Keratinized gingiva ≥ 5 mm	IT ≥ 35 N cm IT ≥ 35 N cm	20 20	0 0	100 100	NR NR
Van Nimwegen et al. [116]	Type 1A	Intact facial bone wall on preoperative CBCT	IT ≥ 45 N cm	60	0	100	NR
Raes et al. [114]	Type 1A vs. Type 4A	Fresh extraction sockets with intact buccal bone wall	IT ≥ 25 N cm	39	1	97.4	Buccal bone dehiscence or fenestration during surgery
Khazaei et al. [117]	Type 1C vs. Type 4C	An adequate mesiodistal space (6.5 mm or more) for placement of an implant with 3.5 mm or maximum possible diameter based on the anatomy of the area	NR	26	0	100	NR
Daher et al. [66]	Type 4A vs. Type 4C	Bone height ≥ 8 mm, bone width allowing implant placement without bone augmentation procedures	NR	NR	NR	NR	NR
Nicolau et al. [118]	Type 4A vs. Type 4B	No need for major bone grafting or ridge augmentation	NR	64	56	87.5	Protocol deviations (eligibility criteria not fulfilled; restorative protocol; incomplete data)
Rattanapanich et al. [119]	Type 2‐3A vs. Type 4C	Bone height ≥ 12 mm, bone width ≥ 6 mm; Keratinized mucosa width ≥ 4 mm	IT > 30 N cm	50	0	100	NR
Merli et al. [120]	Type 1A vs. Type 1C	Adequate bone to place at least one 9.5 mm long implant; Bone width ≥ 5.5 mm	IT ≥ 40 N cm	60	0	100	NR
Wang et al. [125]	Type 4C Type 1A vs. Type 1C	NR NR	NR IT of 30 N cm	38 38	0 NR	100 NR	NR NR
Felice et al. [122]	Type 1C vs. Type 2–3C vs. Type 4C	NR	IT > 35 N cm	NR	NR	NR	NR
Wang et al. [124]	Type 2–3A vs. Type 2–3C	NR	NR	52	0	100	NR
Lee et al. [121]	Type 1A vs. Type 2–3B	Type 1: fresh extraction sockets with intact socket walls Type 2–3: Early placement after 8 weeks of healing	IT ≥ 35 N cm	20	0	100	NR
Slagter et al. [126]	Type 1A and Type 1C	Sufficient bone on the palatal side to place an implant	NR	35	0	100	NR
Linkevicius et al. [127]	Type 4B vs. Type 4A	Bone height ≥ 10 mm, bone width ≥ 6 mm	IT ≥ 35 N cm	59	0	100	NR
Slagter et al. [126]	Type 1A vs. Type 1C	Sufficient bone on the palatal side to place an implant with primary stability, sufficient mesiodistal width for implant placement, excluded if vertical bony defect of 5 mm or more of the labial socket after extraction	NR	40	0	100	NR
Gjelvold et al. [196]	Type 2–3A vs. Type 2–3C	No need for bone grafting or ridge augmentation	IT ≥ 30 N cm	50	0	100	NR
Galindo‐Moreno et al. [128]	Type 4B	Need for bone grafting at time of implant placement	NR	34	3	91.1	Insufficient primary stability
Testori et al. [129]	Type 4A vs. Type 4B	No need for bone grafting or ridge augmentation	IT ≥ 30 N cm for single implants and IT ≥ 20 N cm for splinted implants	52	0	100	NR
Albertini et al. [130]	Type 2–3A vs. Type 2–3C	Adequate bone to place implant with ≥ 8 mm length and ≥ 3.5 mm width, i.e., bone height ≥ 9 mm, bone width ≥ 6.5 mm. Keratinized mucosa buccolingual ≥ 4 mm	No vertical movement	21	0	100	NR
Ky et al. [132]	Type 4A vs. Type 4C	Bone height 11–14 mm height, bone width 6–10 mm; No systemic conditions	IT ≥ 35 N cm	14	0	100	NR
Puisys et al. [131]	Type 1A	NR	IT 25 N cm	50	0	100	NR
Grandi et al. [133]	Type 4A vs. Type 4B	Adequate bone to place implant with ≥ 10 mm length and ≥ 3.7 mm diameter, no need for bone augmentation	IT ≥ 30 N cm	80	0	100	NR
Strasding et al. [134]	Type 1B vs. Type 2–3B	Bone quality and quantity adequate for one‐stage implant placement. Bone height at least 1 mm longer than the length of the implant. Keratinized mucosa at least 2 mm	NR	60	0	100	One early implant failure before restoration delivery
Fawzy et al. [135]	Type 4A vs. Type 4C	Adequate ridge dimensions to receive regular implant, thin gingival phenotype	IT of 25 N cm	20	0	100	NR
CCT							
Achilli et al. [136]	Type 4A vs. Type 4B	NR	IT of 30 N cm	NR	NR	NR	NR
Schropp and Isidor [137]	Type 2–3C vs. Type 4C	NR	NR	NR	NR	NR	NR
Mertens and Steveling [138]	Type 1A vs. Type 4A vs. Type 1B vs. Type 4B	No signs of inflammation; adequate vertical bone height to retain an implant	Good bone quantity and quality; high primary implant stability	NR	NR	NR	NR
De Bruyn et al. [139]	Type 1A vs. Type 4A	No need for bone grafting or ridge augmentation	IT 15–20 N cm	157	44	72	Bone grafting required for implant placement; insufficient primary implant stability
Heinemann et al. [140]	Type 1C vs. Type 4C	NR	NR	NR	NR	NR	NR
Vandeweghe et al. [141]	Type 1A vs. Type 4A	No signs of periapical inflammation	IT ≥ 40 N cm	NR	NR	NR	NR
Meizi et al. [142]	Type 1A vs. Type 4A vs. Type 1C vs. Type 4C	Adequate bone height ≥ 8 mm; Adequate bone width to retain ≥ 1 mm of cortical bone on the buccal and lingual/palatal after osteotomy preparation	IT ≥ 30 N cm	NR	NR	NR	NR
Ormianer et al. [143]	Type 1C vs. Type 2–3C vs. Type 4C	Immediate: no significant infection or pathology. No sinus elevation, bone augmentation, or other advanced procedures were required. Adequate bone volume Early: 6–8 weeks post‐extraction	NR	169	0	100	NR
Jokstad et al. [144]	Type 4C	NR	NR	20	0	100	NR
Ayna et al. [145]	Type 4A vs. Type 4C	Bone height 6.5–8 mm, bone width > 8 mm; No need for bone grafting or ridge augmentation	IT ≥ 35 N cm	63	0	100	NR
Salina et al. [146]	Type 4C	The two implant sites could be adjacent and had to allow the placement of two implants ≥ 6.5 mm long and ≥ 3.4 mm wide, leaving ≥ 1 mm of bone around the implant.	NR	60	0	100	NR
Menchini‐Fabris et al. [147]	Type 1A vs. Type 1C	Fresh extraction sockets with intact socket walls and no dehiscence or fenestration	IT ≥ 35 N cm	54	0	100	NR
Prati et al. [148]	Type 1C vs. Type 2–3C vs. Type 4C	Type 1: hopeless teeth that were free from infection or teeth affected by granuloma with sufficient residual bone Type 2–3: Early placement after 8–12 weeks of healing	NR	66	0	100	NR
Berberi et al. [149]	Type 1A vs. Type 4A	NR	NR	36	0	100	NR
Amato et al. [150]	Type 1C vs. Type 1A	Atraumatic tooth extraction, ensuring that the alveolar plates were preserved	IT ≥ 50 N cm	112	0	100	NR
Retrospective cohort study							
Boronat et al. [180]	Type 1B vs. Type 4B	NR	NR	NR	NR	NR	NR
Belser et al. [197]	Type 2–3B	NR	NR	NR	NR	NR	NR
Blus and Szmukler‐ Moncler [152]	Type 1A vs. Type 1B vs. Type 1C	No signs of periodontal disease or infection at the apex; nonresorbed buccal wall	NR	NR	NR	NR	NR
Becker et al. [154]	Type 1A	≥ 3 mm of apical circumferential bone to place a 5.8 mm or longer implant; ≥ 1 mm inside facial plate	IT ≥ 15 N cm ISQ ≥ 50	NR	NR	NR	NR
Brown and Payne [155]	Type 1A	Presence of 4 mm bone apical to the socket; stable socket walls post‐extraction with three‐wall dehiscence of < 4 mm; sockets allowing to place a 4 × 13 mm or larger implant; Mesial distal proximal distance ≥ 6 mm; adequate bone quality and quantity (Types I–III)	IT 35–40 N cm	27	2	92.6	Insufficient primary implant stability
Fugazzotto [186]	Type 1C	Buccal alveolar wall was intact, or a fenestration was present that was ≥ 5 mm apical to the alveolar crest	NR	NR	NR	NR	NR
Mura [157]	Type 1 A	No signs of active periodontal disease	IT ≥ 45 N cm for single implant; IT ≥ 35 N cm for multiple splinted implants	NR	NR	NR	NR
Hartlev et al. [158]	Type 1A	Marginal bone loss < 1 mm buccally after tooth extraction; no acute infection	IT > 30 N cm	NR	NR	NR	NR
Mangano et al. [159]	Type 1A vs. Type 4A	Intact socket walls; thick gingival biotype; no active periodontal infections; no need for hard/soft tissue grafting before implant placement	NR	NR	NR	NR	NR
Paul and Held [160]	Type 1A	NR	NR	NR	NR	NR	NR
Kolerman et al. [167]	Type 1A	Compromised buccal plate (< 1 mm, dehiscenced or fenestrated); augmentation procedure needed; ≥ 5 mm of apical or palatal bone	IT ≥ 32 N cm	NR	NR	NR	NR
Van Nimwegen et al. [168]	Type 1A	No significant soft tissue loss; distance of the contact point to bone level at the adjacent teeth ntct; midbuccal vertical bone loss ≤ 3 mm	IT > 35 N cm	NR	NR	NR	NR
Sener‐Yamaner et al. [216]	Type 4B	NR	NR	NR	NR	NR	NR
Bonnet et al. [171]	Type 1A	Atraumatic extraction with preservation of intact buccal plate or no more than 2 mm of bone loss. Primary stability achieved during placement.	IT ≥ 30 N cm	39	0	100	NR
Dimaira et al. [192]	Type 1C	Required the explantation of failed implants with removal of fibrous tissue and preparation of the osteotomy to ensure primary stability (IT ≥ 30 N cm)	NR	14	0	100	NR
Menchini‐Fabris et al. [178]	Type 1A	Type 1 extraction socket with fully intact bony walls. Atraumatic flapless tooth extraction and implant placement at least 4 mm beyond the root apex	NR	20	0	100	NR
Prospective cohort study							
Mayer et al. [181]	Type 1C vs. Type 4C	≥ 1 mm of bone available at the buccal and lingual aspects of the implant and below the apex	NR	NR	NR	NR	NR
Romeo et al. [219]	Type 4C	Adequate bone volume	No signs of peri‐implant inflammation	NR	NR	NR	NR
Prosper et al. [182]	Type 1C	Post‐extraction pocket with 4 walls and minimal bone resorption; 3–5 mm of bone below the implant apex	NR	NR	NR	NR	NR
Bianchi and Sanfilippo [183]	Type 1C	Adequate width and height to place an immediate implant	NR	NR	NR	NR	NR
Luongo et al. [201]	Type 4A	Adequate bone volume	IT ≥ 15 N cm	45	5	88.9	Patient withdrawal; insufficient primary implant stability; protocol deviations (multiple implants in one patient)
De Rouck et al. [151]	Type 1A	Ideal soft tissue contour at the facial; normal to thick‐flat gingival biotype; adequate bone height apical to the alveolus of the failing tooth (≥ 5 mm)	IT ≥ 35 N cm	32	2	93.75	Buccal bone dehiscence
Ostman et al. [202]	Type 4A	Adequate bone to place two 7‐mm or longer implants or one 15 mm long implant; no sigh of infection	IT ≥ 30 N cm ISQ ≥ 60	91	14	84.6	Insufficient primary implant stability
Siddiqui et al. [203]	Type 4A	Adequate bone to place a 3.7 mm × 10 mm or larger implant; adequate bone width to preserve ≥ 1.0 mm of buccal and lingual plate thickness after osteotomy preparation	IT ≥ 30 N cm	60	16	98.0	Insufficient bone availability for implant placement
Del Fabbro et al. [184]	Type 1C	Adequate quality and quantity of native bone to achieve primary stability	NR	NR	NR	NR	NR
Bornstein et al. [212]	Type 4B	NR	Bone densities of Type I to III	56	2	96.4	Insufficient implant stability
Valentini et al. [153]	Type 1A	NR	IT ≥ 40 N cm	94	51	45.7	Insufficient primary implant stability
Calandriello and Tomatis [204]	Type 4A	Adequate bone height to place a 8.5 mm or longer implant; implant to crown length ratio ≥ 1:1	IT ≥ 35 N cm	NR	NR	NR	NR
Degidi et al. [205]	Type 4A	Adequate quantity of bone in the surgery site	IT ≥ 25 N cm ISQ ≥ 60	NR	NR	NR	NR
De Angelis et al. [185]	Type 1C	Residual buccal bone to implant gap ≥ 1 mm	NR	95	15	84.2	NR
Fung et al. [206]	Type 4A	Adequate bone height to place a 8.5 mm or longer implant; adequate bone width, no need for bone augmentation	IT ≥ 20 N cm ISQ ≥ 60	NR	NR	NR	NR
Kan et al. [156]	Type 1A	Intact labial bony plate; adequate bone to place a 3.5 × 13.0 mm or larger implant without bone grafting; adequate and harmonious gingival architecture	Adequate primary implant stability	NR	NR	NR	NR
Karabuda, Abdel‐Haq, and Arisan [213]	Type 4B	NR	NR	22	0	100	NR
Covani et al. [187]	Type 1C	≥ 4 mm native bone apical to the root apex; adequate quality	NR	115	17	85.2	Patient withdrawal; insufficient bone availability for implant placement
Oyama et al. [207]	Type 4A	Adequate bone to place a 3.0 × 11 mm or larger implant	IT ≥ 25 N cm	NR	NR	NR	NR
Akca et al. [214]	Type 4B	Adequate bone height to place a 10 mm or longer augmentation to place a regular diameter implant	NR	NR	NR	NR	NR
Buser, Chappuis, Bornstein et al. [198]	Type 2–3C	NR	IT ≥ 35 N cm ISQ < 60	NR	NR	NR	NR
Chappuis et al. [10]	Type 2–3C	NR	NR	20	0	100	NR
Malchiodi et al. [161]	Type 1A	Normal or thick soft tissue biotype; ≥ 2 mm attached keratinized tissue	NR	NR	NR	NR	NR
Covani et al. [188]	Type 1C	Extraction sites with no deficiency of buccal bone plate	NR	NR	NR	NR	NR
Grandi et al. [162]	Type 1A	Adequate bone to place a 3.7 × 11.5 mm or longer implant	IT ≥ 45 N cm	28	3	89.3	Buccal bone dehiscence
Lang et al. [208]	Type 4A	Adequate bone to place a 3.7 to 4.7 × 13 mm or larger implant without grafting; ≥ 2 mm attached keratinized tissue	IT ≥ 35 N cm	NR	NR	NR	NR
Noelken et al. [163]	Type 1A	NR	IT ≥ 15 N cm	NR	NR	NR	NR
Montoya‐Salazar et al. [189]	Type 1C	Adequate quality and quantity of native bone; adequate mesiodistal space (> 7 mm)	Primary implant stability	NR	NR	NR	NR
Calvo‐Guirado et al. [164]	Type 1A	At least 3 mm of bone height and enough width for 4.1 implant diameters	NR	53	0	100	NR
Cristalli et al. [165]	Type 1A	Absence of active infection around the surgical site; adequate bone (≥ 4 mm beyond the root apex); keratinized tissue ≥ 2 mm	IT ≥ 35 N cm	28	4	85.7	Buccal bone dehiscence or fenestration
Migliorati et al. [166]	Type 1A	Adequate native bone; ≥ 2 mm facial keratinized gingiva; Intact facial socket walls or only small dehiscence defects affecting the crestal bone < 3 mm in height	Primary implant stability	48	0	100	NR
Maiorana et al. [215]	Type 4B	NR	NR	69	0	100	NR
Barone et al. [220]	Type 4C	No need for bone grafting or ridge augmentation	NR	120	4	96.7	Bone grafting required for implant placement; patient withdrawal; excessive IT
Meloni et al. [221]	Type 4C	Residual bone height ≥ 10 mm; Residual bone width ≥ 6 mm; ≥ 2 mm keratinized gingiva crestally	IT 35–45 N cm	18	0	100	NR
Fugl et al. [209]	Type 4A	Adequate bone to place a 3.5 × 8 mm or larger implant; no need for major bone augmentation	NR	NR	NR	NR	NR
Noelken et al. [169]	Type 1A	NR	NR	NR	NR	NR	NR
Zuiderveld et al. [62]	Type 1A	Buccal socket wall with bony defect of < 5 mm in vertical direction	NR	60	0	100	NR
Velasco Ortega et al. [170]	Type 1A	Atraumatic extraction of the tooth Integrity of the vestibular bone plate Presence of ≥ 5 mm of bone beyond the root apex to ensure primary stability Absence of diffuse periapical lesions or severe pathology	IT ≥ 35 N cm	56	0	100	NR
Esposito et al. [195]	Type 4C	Sufficient bone volumes for implants of at least 8.5 mm long and 4.0 mm wide.	IT ≥ 35 N cm	30	0	100	NR
Minichetti et al. [210]	Type 4A	NR	IT of 30 N cm	24	0	100	NR
Koutouzis et al. [172]	Type 1A	Sufficient bone volume for implant with 3.5 mm width and 9 mm length without need for bone augmentation	NR	28	1		Not reported
Peñarrocha‐Oltra et al. [190]	Type 1C	Presence of sufficient bone to place a single implant with a length of at least 10 mm and diameter of 3.3 mm. Patients were excluded if the implant could not be placed due to insufficient bone volume or active infection at the extraction site	NR	20	0	100	NR
Peñarrocha et al. [190]	Type 1A	Sufficient bone was required to allow the placement of one single implant at least 10 mm long with a 3.3‐mm diameter	NR	20	0	100	100
Chan et al. [191]	Type 1C	NR	IT ≥ 30 N cm	NR	NR	NR	NR
Jonker et al. [199]	Type 2–3C	GBR group: buccal bone defect ≤ 4 mm; control: implant surface completely in native bone	NR	75	3	96	Patient withdrawal; protocol deviation (differing placement protocol)
Esposito et al. [223]	Type 4C	If 2 implants adjacent to each other planned: enough bone for implants with at least 6.5 mm length and at least 3.4 mm width leaving at least 1 mm bone around implant, no need for bone augmentation at implant placement	NR	60	0	100	NR
De Bruyckere et al. [200]	Type 2–3C	Bucco‐palatal loss of tissue with normal apicocoronal ridge height. Bucco‐palatal bone dimension at least 6 mm at the central and crestal aspect, no need for horizontal bone augmentation at time of implant placement	NR	42	0	100	NR
Waller et al. [224]	Type 4C	Small buccal vertical bone dehiscence (5 mm or less)	NR	22	0	100	NR
Kahramonoglu et al. [225]	Type 4C	NR	NR	30	6	80	Patient withdrawal
Meloni et al. [226]	Type 4C	Residual bone height ≥ 10 mm Residual bone width ≥ 6 mm, with at least 5 mm of keratinized gingiva	IT ≥ 35 N cm	18	0	100	NR
Spinato et al. [222]	Type 4C	Bone crest with at least 6 mm of width and 9 mm of height above the mandibular canal, without concomitant or previous bone augmentation procedures	NR	80	0	100	NR
Shi et al. [193]	Type 1C	Patients with less than 2 mm buccal plate dehiscence	NR	44	1	97.7	Patient withdrawal
Esposito et al. [227]	Type 4C	Bone volume to place 2 implants with at least 8 mm length and 3.75 mm width, no need for tissue augmentation at implant placement	NR	31	5	83.8	Protocol deviations (two adjacent implants; restorative protocol)
Azaripour et al. [174]	Type 1A	Sufficient bone for implant with at least 8.5 mm length and at least 3.75 mm width. At least 4 mm bone apically to tooth apex. If missing buccal bone: after implant placement horizontal space of at least 3 mm between buccal side of implant and adjacent buccal bone	IT of 30 N cm	20	0	100	NR
Gulje et al. [50]	Type 4B	Must be able to receive 1 mm long and 4 mm wide implant	NR	95	1	98.9	Insufficient bone availability for implant placement
Cooper et al. [211]	Type 4A	Minimal 5.5 mm buccolimgual dimension, 5.5 mm distance between adjacent teeth. Keratinized midbuccal height of at least 2 mm	NR	141	0	100	NR
Garcia‐Sanchez et al. [194]	Type 1C	Intact buccal plate, adequate mesiodistal space (at least 1.5 mm bone on each side)	IT of 30 N cm	28	0	100	NR
Levine et al. [217]	Type 4B	A minimum of 3 months post‐extraction healing Adequate ridge height, width, and mesiodistal space for a 4.1‐ or 4.8‐mm wide implant with a minimum length of 8 mm	IT ≥ 35 N cm	22	1	95.5	Lost to follow‐up
Ferrantino et al. [173]	Type 1A	Presence of at least 2 mm of bone apical to the alveolar socket, as assessed in the preoperative CBCT scan	IT > 35 N cm	60	1	98.4	Insufficient primary stability
Salem et al. [218]	Type 4B	Sufficient bone volume for implant with 3.7 mm width and 10 mm length without need for bone augmentation	NR	30	0	100	NR
Siegenthaler et al. [228]	Type 4C	NR	NR	47	0	100	NR
Meijndert et al. [229]	Type 4C	Sites needed to have sufficient bone volume for implant placement without prior alveolar ridge preservation.	NR	30	0	100	NR
Carosi et al. [175]	Type 1A	Extraction sockets with an intact buccal wall and adequate bone quantity to place an implant with a diameter of at least 3.5 mm and a length of at least 10 mm	IT ≥ 35 N cm	40	0	100	NR
Venkatraman et al. [177]	Type 1A	Intact labial periodontal tissue, thick gingival phenotype, at least 3‐5 mm apical bone available	IT ≥ 35 N cm	22	0	100	NR
Todescan et al. [179]	Type 1A	Atraumatic flapless extraction Adequate bone volume to allow for: Implant dimensions of 3.5 × 13.0 mm or greater A minimum of 2 mm between the implant and the internal surface of the facial bone wall A minimum of 1.5 mm between the implant and the proximal aspects of adjacent teeth	IT ≥ 35 N cm	26	8	76	Buccal bone dehiscence; insufficient primary implant stability
Stacchi et al. [230]	Type 4C	Bone crest width ≥ 6 mm at implant site and an available bone height ≥ 9 mm at implant site	NR	51	0	100	NR
Azaripour et al. [176]	Type 1A	Sufficient bone for implant with at least 8.5 mm length and at least 3.75 mm width. At least 4 mm bone apically to tooth apex. If missing buccal bone: after implant placement horizontal space of at least 3 mm between buccal side of implant and adjacent buccal bone	IT ≥ 30 N cm	20	0	100	NR

Abbreviations: ISQ, implant stability quotient; IT, insertion torque; NR: not reported.

#### 3.3.1 Anatomic Criteria for Implant Placement Protocol

An adequate bone height and width for implant placement remains a key requirement for inclusion across studies; however, variations in defining adequacy persist. Most studies do not require bone grafting, and only a few detail specific dimensional requirements. For example, some studies require bone thickness of at least 5.5 mm [120] or 6 mm [115, 211]. Bone height requirements also vary, ranging from 10 mm for residual ridge height [226] to a minimum of 7 mm in specific cases [113].

Studies such as Puisys et al. [131] have emphasized intact buccal bone walls, with specific criteria like allowing Class I extraction sockets or intact facial plates as visualized on CBCT scans [116]. Extraction sockets with partial defects (e.g., dehiscences or fenestrations) are sometimes permissible within strict limits, such as < 4 mm buccal dehiscence [126] or an apical buccal gap of at least 3 mm between implant and adjacent bone [176]. Additional criteria, such as sufficient keratinized gingiva (≥ 5 mm) and minimally traumatic extraction techniques, are common among newer studies.

#### 3.3.2 Procedural Criteria for Implant Loading Protocol

Primary implant stability continues to be a central criterion for immediate and early loading. The most commonly reported measure is implant insertion torque (IT). Thresholds vary among studies, ranging from IT ≥ 25 N cm [131, 135] to IT ≥ 40 N cm [115, 120]. In some cases, IT ≥ 35 N cm with no adverse clinical symptoms is necessary for immediate restoration [150]. Immediate loading is also supported by high resonance frequency analysis values, such as ISQ ≥ 60, as reported in Wang et al. [124] and Meloni et al. [226]. Cases requiring delayed loading often involve IT values < 35 N cm or bone densities unsuitable for immediate functional load.

Other notable procedural criteria include adequate buccal‐lingual bone width (≥ 6 mm, [119, 211]) and a vertical dimension exceeding the implant length by at least 1 mm [134]. Studies such as Todescan et al. [179] and Levine et al. [217] emphasize the importance of precise preoperative planning and atraumatic surgical techniques to ensure optimal primary stability.

No study reported occlusal scheme as an exclusion criterion, although all applied functional loading and emphasized controlled occlusion and absence of parafunction.

Using the validation criteria tool for the 12 types of placement and loading protocols, Type 1A, Type 2–3A, Type 4A, Type 4B, Type 1C, Type 2–3C, and Type 4C were SCV. Type 1B and Type 2–3B were clinically documented (CD) (Table 15).

**TABLE 15:** Scientific and clinical validation according to the implant placement and loading protocol.

	Loading protocol		
	Immediate restoration/loading (Type A)	Early loading (Type B)	Conventional loading (Type C)
Implant placement protocol			
Immediate placement (Type 1)	Type 1A SCV^a^	Type 1B CD^a^	Type 1C SCV^a^
Early placement (Type 2–3)	Type 2‐3A SCV^a^	Type 2‐3B CD^a^	Type 2‐3C SCV
Late placement (Type 4)	Type 4A SCV^a^	Type 4B SCV	Type 4C SCV

*Note:* Type 1A: immediate placement + immediate restoration/loading; Type 1B: immediate placement + early loading; Type 1C: immediate placement + conventional loading; Type 2–3A: early placement + immediate restoration/loading; Type 2–3B: early placement + early loading; Type 2–3C: early placement + conventional loading; Type 4A: late placement + immediate loading; Type 4B: late placement + early loading; Type 4C: late placement + conventional loading. CD (yellow): clinically documented; CWD (green): clinically well‐documented; SCV: scientifically and clinically validated.

^a^ Specific selection criteria for immediate implant placement and immediate loading protocols based on the last ITI Consensus [2, 3].

#### 3.3.3 ITT Analysis

Table 14 summarizes ITT outcomes for updated studies. Across 140 studies reporting ITT, 65 (46.4%) achieved 100% ITT adherence, indicating no divergence between planned and performed treatments, whereas 31 (22.1%) reported < 100% ITT with documented exclusions. The most frequent reported reasons were insufficient primary stability in 9 studies, patient withdrawal/loss to follow‐up in 9, inadequate bone volume/dimensions or a need for grafting procedures in 7, buccal bone dehiscence or fenestration in 6, and protocol deviations (e.g., change in prosthetic plan) in 4 studies. Representative examples include [179] (exclusions due to facial bone fractures during extraction) and [217] (loss to follow‐up). A minority of retrospective series attributed exclusions to poor adherence to predefined inclusion criteria (e.g., inadequate buccal bone preservation; [192]). The remaining studies, 44 (31.5%) failed to report enough information for an ITT analysis.

## 4 Discussion

The relationship between implant placement and loading protocols remains a key consideration in successful treatment planning. Historically, reviews such as those by Buser et al. [9] and Papaspyridakos et al. [13] analyzed these factors separately. However, Gallucci et al. [1, 21] introduced a paradigm shift by advocating for an integral approach, emphasizing their interdependence to optimize clinical outcomes. This conceptual shift also facilitated the development of a bio‐restorative treatment philosophy [231]. This framework systematically integrates biological and restorative principles through digital planning, thereby enhancing implant positioning and esthetic predictability. It also clarifies the need for precise coordination between surgical and restorative phases to improve overall treatment planning.

**FIGURE 2:**
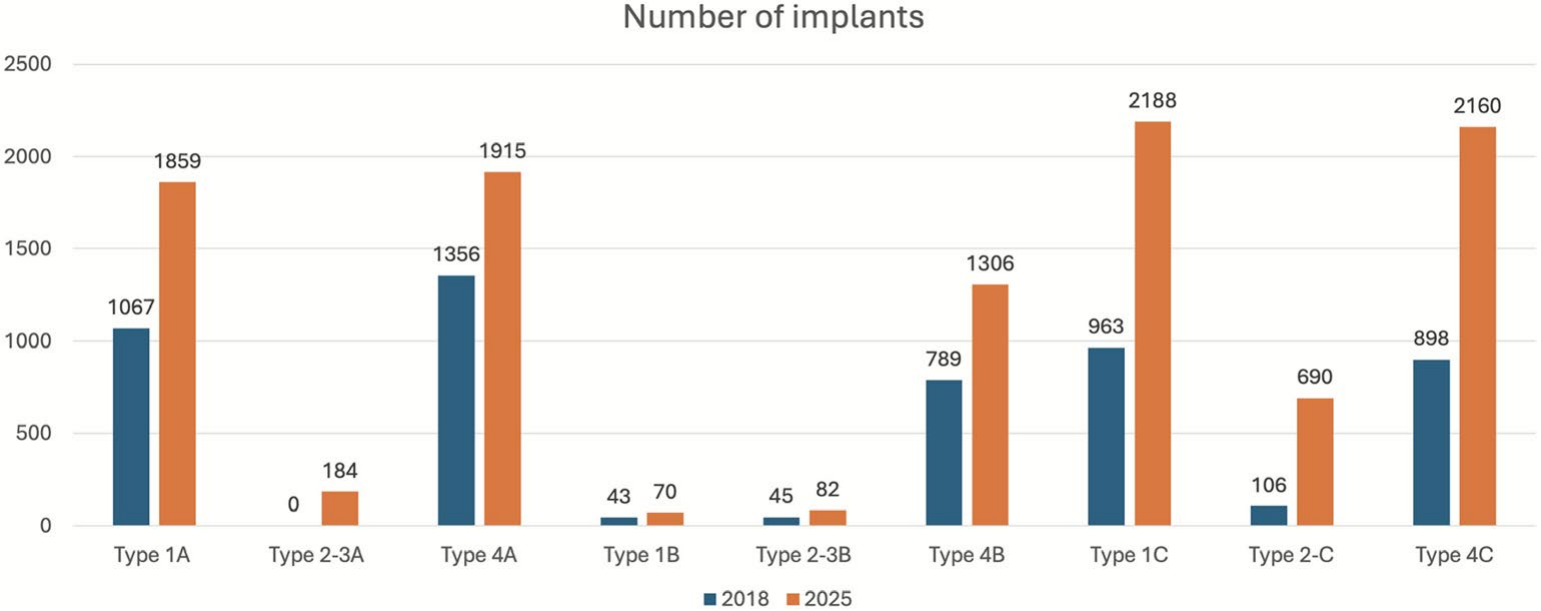
Comparison of protocol outcomes from 2018 to 2025 by number of implants. Type 1A: immediate placement + immediate restoration/loading; Type 1B: immediate placement + early loading; Type 1C: immediate placement + conventional loading; Type 2–3A: early placement + immediate restoration/loading; Type 2–3B: early placement + early loading; Type 2–3C: early placement + conventional loading; Type 4A: late placement + immediate loading; Type 4B: late placement + early loading; Type 4C: late placement + conventional loading.

**FIGURE 3:**
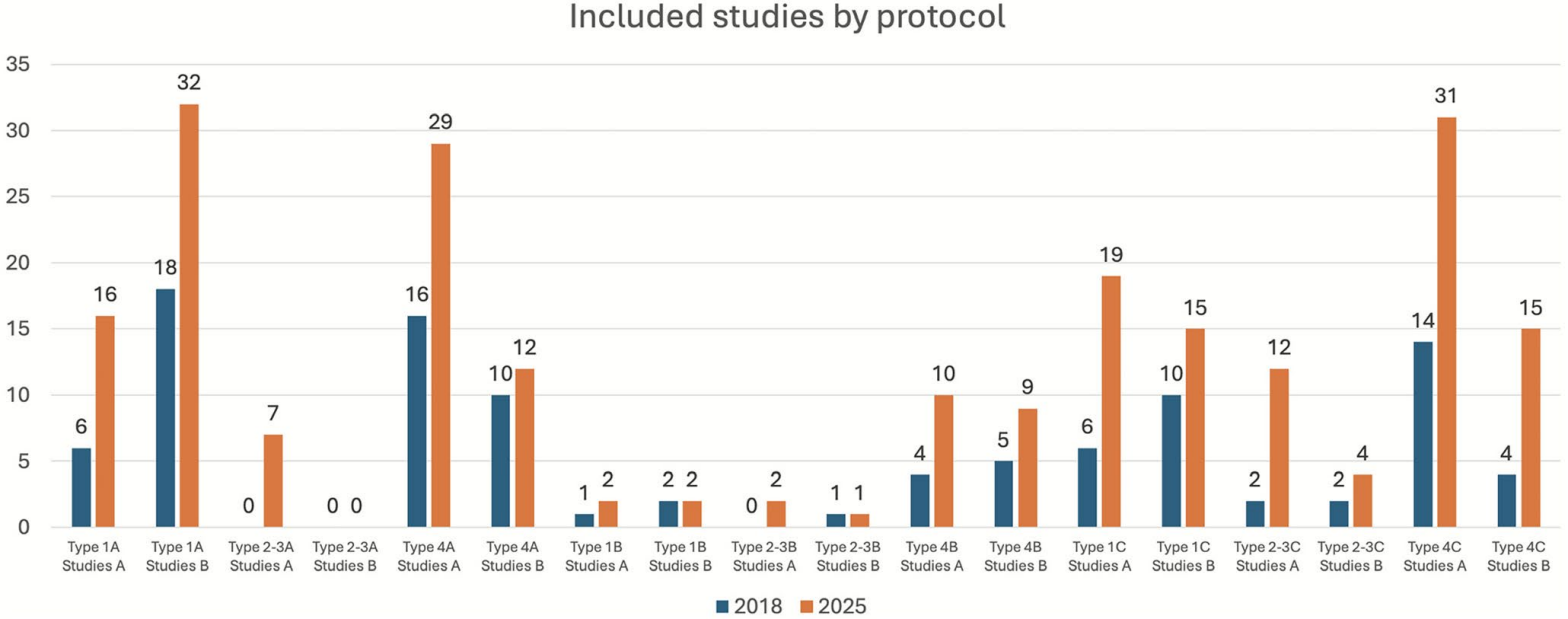
Comparison of protocol outcomes from 2018 to 2025 by study design. Type 1A: immediate placement + immediate restoration/loading; Type 1B: immediate placement + early loading; Type 1C: immediate placement + conventional loading; Type 2–3A: early placement + immediate restoration/loading; Type 2–3B: early placement + early loading; Type 2–3C: early placement + conventional loading; Type 4A: late placement + immediate loading; Type 4B: late placement + early loading; Type 4C: late placement + conventional loading. Studies A: Comparative studies; Studies B: Non‐comparative studies.

This updated review expands on the 2018 findings by incorporating a broader dataset and more extended follow‐up periods (Tables 13 and 16; Figures 2 and 3). For example, the number of implants analyzed under the Type 1A protocol increased from 1079 in 2018 to 2021 in 2024, supported by 17 clinical trials and 35 non‐comparative studies. Recent international consensus conferences further demonstrate an increasing scientific evidence and clinical support for immediate implant placement combined with immediate loading, particularly within the anterior maxilla [4].

**TABLE 16:** Comparison of the outcomes of different protocols from 2018 to 2025.

	Implant loading protocol					
	Immediate restoration/loading (Type A)		Early loading (Type B)		Conventional loading (Type C)	
Implant placement protocol	2018	2025	2018	2018	2025	2018
Immediate placement (Type 1)	Type 1A CD 1067 Implants 6^a^ 18^b^	Type 1A SCV 1859 Implants 16^a^ 32^b^	Type 1B CD 43 Implants 1^a^ 2^b^	Type 1B CD 70 Implants 2^a^ 2^b^	Type 1C SCV 963 Implants 6^a^ 10^b^	Type 1C SCV 2188 Implants 19^a^ 15^b^
Early placement (Type 2–3)	Type 2‐3A CID NA Data	Type 2‐3A SCV 184 Implants 7^a^ 0^b^	Type 2‐3B CID 45 Implants 0^a^ 1^b^	Type 2‐3B CD 82 Implants 2^a^ 1^b^	Type 2‐3C SCV 106 Implants 2^a^ 2^b^	Type 2‐3C SCV 690 Implants 12^a^ 4^b^
Late placement (Type 4)	Type 4A CD 1356 Implants 16^a^ 10^b^	Type 4A SCV 1915 Implants 29^a^ 12^b^	Type 4B SCV 789 Implants 4^a^ 5^b^	Type 4B SCV 1308 Implants 10^a^ 9^b^	Type 4C SCV 898 Implants 14^a^ 4^b^	Type 4C SCV 2160 Implants 31^a^ 15^b^

*Note:* Type 1A: immediate placement + immediate restoration/loading; Type 1B: immediate placement + early loading; Type 1C: immediate placement + conventional loading; Type 2–3A: early placement + immediate restoration/loading; Type 2–3B: early placement + early loading; Type 2–3C: early placement + conventional loading; Type 4A: late placement + immediate loading; Type 4B: late placement + early loading; Type 4C: late placement + conventional loading. CD (yellow): clinically documented; CID (red): clinically insufficiently documented (includes loading protocols that are not documented); CWD (green): clinically well‐documented; SCV: scientifically and clinically validated. Superscripts denote study design: a = comparative; b = non‐comparative.

Previously underreported protocols, such as Type 2–3A and Type 2–3B, now have more extensive scientific and clinical validation. This updated review further reinforces the reliability of the findings and provides a more robust foundation for evaluating the outcomes of different implant placement and loading protocols. At the same time, it underscores the growing adoption of various protocols in clinical practice.

Type 1A demonstrated a slight decrease in cumulative survival rate (98.4% in 2018 to 96.73% in 2024), likely due to a more diverse dataset. The extended follow‐up (up to 120 months) offers further insights into long‐term outcomes and the challenges of maintaining success over time. In 2018, Type 1A was classified as clinically documented (CD) due to insufficient studies. Since then, research has expanded, with analyzed implants rising from 1079 to 2021 and clinical trials increasing from 6 RCTs and 18 non‐comparative studies to 17 RCTs and 35 non‐comparative studies. Initially, the wide variability in survival outcomes (71.43%–100%) warranted retention of Type 1A within CD status. However, the application of refined statistical methods—specifically the exclusion of extreme outliers—now supports its reclassification as SCV.

Certain studies presented results markedly outside the remaining dataset's range. These extreme values were excluded after an in‐depth assessment, indicating that methodological inconsistencies may have unduly influenced the findings. Excluding these outliers preserved the methodological rigor of the pooled analysis and ensured that the synthesized outcomes more accurately represent standard clinical performance. This reclassification underscores this protocol's potential for predictable outcomes under controlled conditions. However, its success remains highly technique‐sensitive, requiring careful case selection, surgical precision, and adherence to prosthetic guidelines. Immediate implant placement and loading, particularly in the anterior maxilla, have demonstrated high survival rates and reduced treatment times, making them attractive options for patients and clinicians [4, 5]. However, optimal outcomes depend on strict adherence to patient selection criteria, including adequate buccal bone thickness, preservation of socket walls, and achievement of sufficient primary stability [2, 3].

Immediate placement with early loading (Type 1B) experienced a notable decline in survival rate (98.2% to 91.6%), raising concerns about its predictability. The limited number of available studies continues to hinder the clinical validation of Type 1B and 2–3B despite reported 91.6% and 100% survival rates, respectively. Type 1B and Type 2–3B introduce higher biological risks without significant benefits over well‐established protocols. Early loading represents a biologically vulnerable period during which the implant transitions from primary stability to secondary osseointegration, thereby elevating the risk of failure. While early loading of delayed implants benefits from osseointegration within healed bone, immediate (Type 1B) and early (Type 2–3B) placements occur under biomechanically less favorable conditions. Immediate placement involves insertion into fresh extraction sockets, whereas early placement occurs within partially healed bone, where residual defects may be present and substantial bone augmentation is frequently required. Type 1A appears to be a more predictable option for reducing surgical interventions and treatment duration, while Type 4C offers a more conservative, safer, biologically favorable alternative. The lack of clear clinical advantages explains the limited adoption and validation of early loading protocols despite available survival data.

Early placement with immediate restoration (Type 2–3A) is supported by validated data, demonstrating a 97.8% survival rate across seven clinical trials. Conventional loading in early implant placement (Type 2–3C) expanded its evidence base, with 12 clinical trials supporting a 94.02% survival rate over a mean follow‐up of 48 months. Early implant placement protocols are bridging the gap between immediate and late placement approaches.

Late placement protocols, including Type 4B and Type 4C, maintained stable outcomes, demonstrating only marginal reductions in survival rates (97.85% and 97.49%, respectively) alongside improved levels of validation. These findings reinforce the suitability of these protocols across diverse clinical contexts. The site of the implant placement and loading protocols seems essential; according to the literature, the Type 4 protocol is very reliable in the mandible, specifically in the posterior region [12]. Type 4 protocol shows higher survival rates when placing implants in the anterior region of the mandible than in immediate implant placement (Type 1) [232]. As this review has shown, Type C loading provides the highest long‐term predictability; the data found in this review is related to partially edentulous rehabilitation. However, according to other reviews, this long‐term predictability is also present in completely edentulous patients [13].

Although some protocols are described as scientifically and clinically validated and report high overall survival rates, the findings suggest that substantial differences in implant survival may exist between them. For example, immediate implant placement with immediate loading (Type 1A) achieved a weighted mean survival of 98.01% (1859 implants, 32 studies). In contrast, the same placement protocol combined with early loading (Type 1B) resulted in a survival rate of 91.62% (70 implants, 2 studies), indicating a four‐fold difference in implant loss. Given the small sample size in Type 1B, one cannot exclude the possibility of a five‐fold difference based on the likely 95% confidence interval. Similarly, for immediate placement under the 2–3 classification, survival ranged from 100.00% (Type 2–3B, 82 implants, 2 studies) to 94.02% (Type 2–3C, 690 implants, 12 studies). These findings underscore the importance of caution when interpreting protocols labeled as “scientifically and clinically validated,” as clinically meaningful differences in survival are possible even when such terminology is used.

Comparison with the broader literature confirms that high implant survival rates can be achieved with various implant placement and loading protocols. For example, the 6th EAO Consensus reported comparable outcomes among immediate, early, and delayed protocols despite inherent study heterogeneity [233]. Similarly, Pommer et al. [234] stated through a meta‐analysis that long‐term outcomes of maxillary single‐tooth implants are not significantly affected by the timing of placement or loading. Another systematic review by Aiquel et al. [235] corroborated these findings by showing that survival rates across various timing protocols for multiple‐unit prostheses are largely comparable.

The heterogeneity in study designs included in this review remains a significant limitation. As in the 2018 analysis, the inclusion of non‐randomized studies introduces potential biases, complicating the interpretation and synthesis of results. ITT analyses would help mitigate this limitation by providing a more accurate representation of how protocol deviations such as modifications in implant placement, inadequate primary stability, or anatomical constraints affect clinical outcomes in real‐world settings. ITT analyses were identified in 96 of the 140 studies, underscoring the need for more standardized methodologies to enhance clinical applicability. Furthermore, specific patient inclusion criteria continue to restrict generalizability, as reported survival rates often reflect outcomes in highly selective patient groups with favorable conditions.

Despite the large number of included studies, the evidence was not amenable to meta‐analysis because of substantial clinical, methodological, and reporting heterogeneity. Clinically, studies combined different placement and loading protocols, implant systems and surfaces, grafting approaches, timing windows, and prosthetic designs, even within single reports. Methodologically, designs ranged from RCTs to CCTs and cohorts with variable risk of bias, with outcomes reported at different analysis levels (implant vs. patient) and across nonaligned follow‐up periods. Reporting was often incomplete (e.g., survival without event counts, confidence intervals, or variance estimates), preventing calculation of a common effect size and its variance. Under these circumstances, statistical pooling would risk imprecise or misleading summary estimates; therefore, a meta‐analysis was not performed.

Survival was the only outcome consistently reported across studies; therefore, complications, esthetic outcomes, and patient‐reported measures could not be quantitatively analyzed. Although all eligible studies were included qualitatively, a small number of statistical outliers were excluded from the weighted calculations to maintain data robustness and prevent disproportionate influence from atypical studies. This methodological decision may introduce minor bias and slightly reduce the representation of small or clinically distinct cohorts; however, it is consistent with Cochrane recommendations aimed at preserving analytical stability in heterogeneous datasets. Additionally, the weighting method applied gives greater influence on studies with longer follow‐up periods, which may marginally affect pooled survival estimates.

These limitations should be considered when interpreting the overall findings and validation outcomes. Future studies should aim to minimize the moderate‐to‐high risk of bias observed across current evidence through well‐designed, prospective randomized trials with standardized protocols, longer follow‐up, blinding, and transparent reporting.

This updated review further solidifies the reliability of the findings and establishes a stronger foundation for assessing the outcomes of various implant placement and loading protocols. It also highlights the increasing clinical adoption of diverse protocols, reflecting evolving trends in treatment approaches. Continued research and standardized reporting are essential for refining protocols and optimizing patient outcomes.

## 5 Conclusion

This review reinforces the importance of evaluating implant placement and loading protocols as an integrated treatment strategy rather than as separate variables. The broadened dataset and extended follow‐up periods have yielded more comprehensive insights into each of the 12 placement/loading types. In immediate placement, Type 1C shows strong survival rates. It is considered scientifically and clinically validated, while Type 1A also meets the criteria for a scientifically and clinically validated protocol with high survival rates. Meanwhile, Type 1B continues to show lower and more variable survival rates—being clinically documented—underscoring the need for careful case selection.

Regarding early placement, Type 2–3C is recognized as a scientifically and clinically validated protocol. Type 2–3A, which was previously underreported, now demonstrates similarly validated survival rates that expand the evidence for early implant placement with immediate loading. Although Type 2–3B is clinically documented, it still lacks sufficient evidence, and further research is necessary to establish its predictability. Finally, all late implant placement protocols are considered scientifically and clinically validated: Type 4C offers high survival with long‐term predictability, while Type 4A and Type 4B maintain stable survival rates backed by well‐established evidence.

## Author Contributions

**German O. Gallucci:** concept/design, data analysis/interpretation, drafting article, critical revision of article. **Adam Hamilton:** concept/design, data analysis/interpretation, critical revision of article. **Samuel Akhondi:** data collection, drafting article, critical revision of article. **Kevser Pala:** data collection, drafting article. **Juan Francisco Peña‐Cardelles:** concept/design, data collection, data analysis/interpretation, drafting article, critical revision of article.

## Funding

The authors have nothing to report.

## Conflicts of Interest

The authors declare no conflicts of interest.

## Data Availability

The data that support the findings of this study are available from the corresponding author upon reasonable request.
